# A multi-objective portfolio optimization model incorporating sentiment analysis of quarterly reports and LSTM-based price prediction

**DOI:** 10.1371/journal.pone.0335036

**Published:** 2026-07-09

**Authors:** Esmaeil Taheripour, Seyed Jafar Sadjadi, Babak Amiri

**Affiliations:** School of Industrial Engineering, Iran University of Science and Technology, Tehran, Iran; Beihang University, CHINA

## Abstract

Sentiment analysis (SA) of natural language text has become as a powerful instrument for enhancing financial market predictions. Quarterly reports from companies, in particular, offer a rich source of data for sentiment analysis, providing key insights into a company’s performance, strategic actions, and future prospects. These reports can significantly influence investor decisions regarding asset investments. Notwithstanding the potential, prior research has not investigated sentiment analysis concerning these resources in portfolio optimization. To fill this void, we propose an innovative three-stage approach to constructing stock portfolios. In the first stage, we perform sentiment analysis on companies’ quarterly reports using the FinBERT model to assess the sentiment surrounding each company. In the second stage, we utilize a Long-Short-Term Memory (LSTM) model for forecasting future prices, which enables the calculation of expected returns and the covariance matrix. In final stage, we present a three-objective portfolio optimization model that incorporates risk, return, and sentiment-derived trend features. We solve this model using the Weighted Goal Programming (WGP) method. Our results indicate that the proposed model effectively supports portfolio optimization. Moreover, the model is implemented using data from companies that are part of the Dow Jones Industrial Average (DJIA), and findings demonstrate high accuracy, confirming the practical potential of the proposed approach.

## 1. Introduction

Wealth management is crucial for aiding people, families, and institutions in safeguarding their financial future, boosting their assets, and mitigating financial challenges. One of the most important aspects of evolving and understanding financial markets has been portfolio optimization and diversification, which has also had a significant impact on decision-making strategies [1]. A significant breakthrough occurred with the publication of modern portfolio selection theory by Markowitz. [2]. Generally, fundamental and technical analysis are two popular procedures for forecasting the future profitability of stocks. Fundamental analysis looks at the underlying economic dynamics of supply and demand, which are the primary mechanisms responsible for driving changes in stock valuations. Conversely, technical analysis scrutinizes previous data concerning price fluctuations and trading volume, employing graphs and indicators as major instruments to forecast future price trends [3,4]. Considering the advancements in computer science and artificial intelligence in recent years, investors can employ these tools to enhance their decision-making. Recent research indicate that artificial intelligence(AI) can outperform traditional portfolio management strategies grounded in classical financial theories, like Modern Portfolio Theory (MPT) [5–7]. In addition, SA has grown in importance in recent years due to advancements in both hardware and software technologies, coupled with the vast availability of financial data.

As regards the swift expansion of Internet-based applications, including social media platforms and blogs, has led to an increase in comments and reviews regarding daily activities. A substantial volume of information obtained from the markets today enables investors to promptly modify their plans as needed. Sentiment analysis of natural language statements can improve predictions in financial markets. Numerous investors depend on information derived from newspapers or their emotions [8]. SA is a continually evolving area of research within the domain of text mining. SA is the computer study of opinions, attitudes, and subjectivity throughout text [9]. SA involves the collection and examination of individuals’ ideas, thoughts, and perceptions of diverse themes, products, issues, and services [10]. SA models categorize sentences (or complete texts) according to their sentiment (positive, negative, or neutral) and generate a sentiment score. Given that the area of SA has garnered the focus of numerous scientists in recent years, they have provided a variety of models and methodologies. SA is an essential application of natural language processing (NLP) focused on detecting and extracting emotions and subjective information from text. Natural languages are those languages utilized by humans for communication. NLP encompasses all the components required for a computer to comprehend and generate natural language. NLP is an area of Artificial Intelligence and linguistics, dedicated to enabling computers to comprehend assertions or phrases articulated in human languages [11]. Since the introduction of the Transformer architecture by Vaswani and colleagues in 2017 their work “Attention is All You Need,” [12] significant advancements have occurred in NLP, resulting in the development of several models, including the BERT [13] family and GPT [14], which have been trained on extensive datasets. Transformer-based models unlike to conventional models like RNN [15], LSTM, and GRU [16] that handle data sequentially from left to right, transformers process all inputs simultaneously. This investigation utilizes a pre-trained model, FinBERT [17], derived from the BERT architecture, for sentiment analysis of textual data. FinBERT is a pre-trained NLP model specifically designed to evaluate the sentiment expressed in financial documents. The BERT language model is enhanced through further training in the finance sector, utilizing a substantial financial corpus to optimize it for financial sentiment categorization.

A significant hurdle in portfolio optimization lies in accurately forecasting expected returns. Anticipating stock market trends is especially challenging in time series analysis because of its complex, nonlinear, dynamic, and often noisy characteristics, making it inherently unpredictable [18]. In reality, stock prices are affected by multiple variables, notably political events, corporate policies, news, and economic factors, interest rates, and investor feelings [19,20]. Incorporating return forecasting from conventional time series models into portfolio construction can enhance the efficacy of the first portfolio optimization model. Given that machine learning and deep learning models have demonstrated considerable superiority over time series models, these methodologies can be employed to enhance outcomes [21]. Recently, numerous researchers have employed various machine learning models for stock market prediction, yielding satisfactory results, including support vector regression (SVR) [22–24] and RF [25,26]. Artificial neural networks (ANNs) [27] As the core principles of deep learning technology have been widely utilized in stock market prediction. One of these approaches is the LSTM model that was presented by Hochreiter & Schmidhuber [28]. LSTM networks are a novel approach for sequence learning [29]. Unlike traditional recurrent neural networks (RNNs), LSTM networks are specifically designed to capture long-term dependencies in time series data. LSTM models utilize a unique architecture with memory cells and control mechanisms, including input, output, and forget gates, which allow them to store and manage important information over time. As a result, this article applies the LSTM model to predict stock returns.

This study introduces a three-stage model for long-term (quarterly) investment decision-making. In the first stage, we analyze available textual data to forecast future price trends. The second stage involves predicting the expected return for the upcoming quarter using a deep learning approach based on LSTM networks. Finally, in the third stage, we determine the optimal asset allocation strategy to maximize potential returns. The main contribution of this model lies in its integrated framework, which combines text-based forecasting, deep learning, and portfolio optimization to support more informed investment decisions. Overall, the key innovations of the proposed model can be summarized as follows:

- Quarterly corporate reports will be utilized to forecast stock price movements. After retrieving these reports from Yahoo Finance, the FinBERT model and Sider AI tool will be employed to analyze their content and assess the market outlook for the forthcoming quarter.
- To identify the ideal asset combination, we provide a mathematical model comprising three objective functions: risk, return, and trend attractiveness.
- The weighted goal programming (WGP) technique is used to solve the three-objective model proposed in this paper.
- The proposed model has been implemented using real-world data from companies listed in the Dow Jones Industrial Average (DJIA), and its results have been empirically demonstrated.

The remainder of this paper is structured as follows: Section 2 provides a review of the related literature, focusing on sentiment analysis, machine learning approaches for stock market forecasting, and portfolio optimization techniques. Section 3 presents the proposed model, detailing its sequential components and offering an overview of key concepts such as FinBERT, LSTM, and the mean-variance framework. Section 4 develops the formal model, describes the proposed solution methodology, and establishes the complete mathematical formulation. Section 5 outlines the data collection process and reports the empirical results obtained from applying the model. Section 6 offers a critical analysis and interpretation of the findings. Finally, Section 7 concludes the study by summarizing the main insights and proposing directions for future research.

## 2. Related work and theories

This section reviews prior research across three key domains relevant to our study. First, we examine sentiment analysis techniques, particularly those tailored for financial texts, which have evolved significantly with the advent of transformer-based models like FinBERT. These tools have enabled more accurate extraction of investor sentiment from unstructured financial documents. Second, we explore machine learning models used for price prediction and forecasting expected returns, highlighting how algorithms such as neural networks, support vector machines, and ensemble methods have improved predictive performance in financial markets. Finally, we review existing frameworks for optimal asset allocation, with a focus on both classical models such as mean-variance optimization.

### 2.1. Portfolio formation

MPT was founded when Markowitz [2] proposed the mean-variance (MV) approach as a solution to the portfolio selection problem, using expected return to measure investment performance and variance to quantify risk. The primary concept of MV technique is to optimize anticipated return while maintaining constant variation, or to minimize variance while preserving expected return. MPT has garnered extensive acceptance and discussion by scholars [5,30]. According to Tobin [31], liquidity preference may influence the amount of wealth allocated to financial assets and creates a successful portfolio that includes both risk-free and a unique kind of risky assets. In order to simplify the computation, Sharpe [32] proposes the diagonal model, which greatly aids in the development of portfolio theory by assuming that there are no relationships between securities. Ritter and Renwick [33] present a comprehensive examination of the literature concerning asset management and investor portfolio behavior, juxtaposing contemporary real-world performance with theoretical advancements, followed by an exploration of their own perspectives on the topic. Other research encompasses Merton’s study [34], which enhanced modern portfolio theory by presenting a continuous-time model aimed at maximizing expected utility within a defined planning framework. To address a real portfolio problem without the need to solve a mathematical programming problem, Elton et al [35] studied and created decision rules. In order to compare MV and quadratic approximations that determine optimal portfolios, Gracer and Hakansson [36] introduced a discrete dynamic investment model. A broad methodology for assessing a managed portfolio’s performance was put forth by Chen and Knez [37]. In order to provide a deterministic transformation to the multi-objective stochastic programming portfolio model, Ben Abdelaziz et al [38] develop a paradigm known as chance constrained compromise programming (CCCP). Several realistic constraints were added to the Markowitz’s MV model by other researchers. Liu and Loewenstein [39] use transaction costs into stock trading methods to optimize investors’ wealth utility. In the MV model, Brown and Smith [40] take into account risk aversion, transaction cost, and portfolio limitations. They conclude that when three additional assets are introduced, portfolio optimization problems will be challenging to resolve. Additionally, several research include robust optimization strategies for portfolio management. In order to estimate uncertain parameters, Tu and Zhou [41] encompass financial objectives into Bayesian priors. They demonstrate that the Bayesian method performs better when using objective-based priors than when using alternative priors for portfolio selection. Fuzzy set theory is also being used by several academics to analyze portfolio issues. According to Li and Xu [42], the financial market frequently experiences both random and fuzzy uncertainty; for this reason, they take into account the opinions of experts and investors and building a portfolio. An expert system is created by Yunusoglu and Selim [43] to assist portfolio managers in making investment decisions. The first step in the three-stage expert method is to remove any merchandise that is deemed unsuitable. The second step involves assessing the stock by conducting in-depth literature searches and speaking with subject-matter experts. Building a portfolio using a mixed-integer linear programming model is the final step. Their findings show that ES performance does not differentiate significantly among risk choices; additionally, ES is better suited for investment periods of six, nine, and twelve months. In order to determine the ideal portfolio, Almahdi and Yang [44] first established three optimization goals: the analytical Sharpe ratio, the Sterling ratio, and the Calmar ratio. Then, they chose the method that performed the best. During the decision-making process, Larni-Fooeik et al. [45] analyzed investment scenarios and determined the quantity of regret attained in each scenario. They evaluated volatility risk measurements and employed stochastic optimization to determine the optimal scenario that optimizes investment portfolio returns, minimizes risk, and reduces resultant regret. The augmented epsilon constraint (AEC) method was employed to transform each multi-objective model into a single objective and to provide the Pareto-efficient frontier. The literature review that was done in the area of portfolio formation is summarized in Table 1 below.

**Table 1:** A summary of the research done in the area of portfolio optimization.

No.	Author	Year	Number of Goals	Risk Criterion	Solution Methods	Transaction Cost	Risk Free Assets	Reference
1	Kellerer et al.	2000	Multi Objective	Semi-Absolute Deviation	Heuristic	Yes	No	[46,47]
2	Rockafeller and Uryasev	2002	Single Objective	CVaR	-	No	No	[48,49]
3	Abdelaziz et al.	2007	Multi Objective	Variance	Stochasting Programming	No	No	[38]
4	Cura	2009	Multi Objective	Variance	PSO Algorithm	No	No	[50]
5	Liu et al.	2012	Multi Objective	Variance	TOPSIS	Yes	No	[51]
6	Liu et al.	2013	Single Objective	Variance	PSO and Crisp Algorithm	Yes	Yes	[52]
7	Gupta et al.	2014	Single Objective	Absolute Mean Deviations	Discrete approximate Iteration Method	Yes	No	[53]
8	Zhang and Liu	2015	Multi Objective	Semi Fuzzy Variance	Genetic Algorithm	Yes	Yes	[54]
9	Vercher and Bermudez	2015	Multi Objective	Semi Absolute Deviation	Genetic Algorithm	No	No	[55]
10	Yao et al.	2016	Single Objective	Variance	Dynamic Programming	No	No	[56]
11	Mehlawat	2016	Multi Objective	Entropy	Goal programming	Yes	No	[57]
12	Guo et al.	2016	Single Objective	Fuzzy Variance	Genetic Based Fuzzy Simulation	yes	No No	[58]
13	Mei et al.	2016	Single Objective	Fuzzy Variance	-	Yes	No	[59]
14	Wang et al.	2020	Multi Objective	Variance	-	No	No	[60]
15	Vaezi et al.	2020	Single Objective	Risk Preferences of Investors	Discrete firefly algorithm	No	No	[61]
16	Ding and Uryasev	2022	Single Objective	Expected Regret of Drawdown	Exact Solution	No	No	[62]
17	Cacador et al.	2022	Single Objective	Regret	GA	No	No	[63]
18	Kagrecha et al.	2023	Multi Objective	Regret	Con-LCB	No	No	[64]
19	Larni-Fooeik et al.	2024	Multi Objective	Semi Absolute Deviation	Epsilon-constraint	No	No	[45]

### 2.2. *Sentiment analysis*

**Fig 1:**
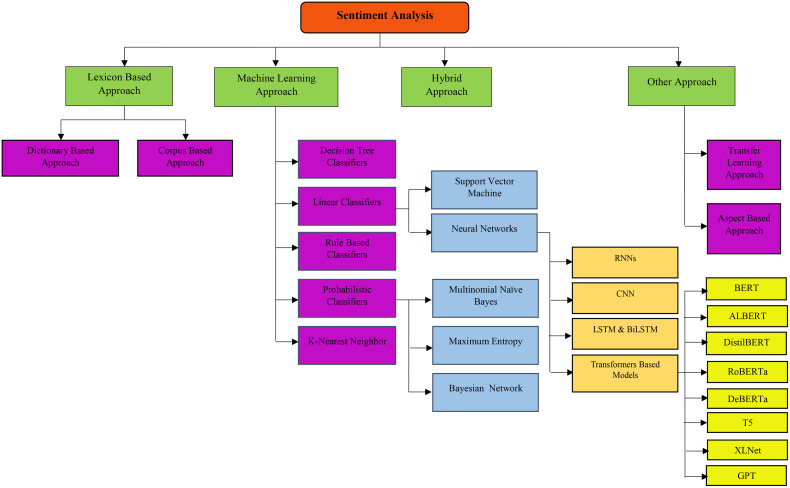
An overview of sentiment analysis algorithms.

The growing interest and applicability of automated sentiment or polarity analysis using NLP has led to the development of various methodologies in recent years. These approaches can be broadly classified into two main categories: lexical-based methods and supervised machine learning methods [5]. Lexical approaches generally employ a predetermined lexicon of words, each linked to a particular sentiment, however they are highly contingent upon the context for which they were developed, as noted by Ribeiro et al [65]. Several instruments such the Valence Aware Dictionary and sEntiment Reasoner (VADER) [66], integrate lexical analysis with the evaluation of phrase features to detect term polarity. Recently, following the introduction of the Transformer conception in 2017 [67] and the subsequent development of models derived from it, particularly Google’s Bidirectional Transformer (BERT) model [68] and its different versions [69,70], have achieved remarkable popularity and established new benchmarks in NLP tasks within the GLUE (General Language Understanding Evaluation) Benchmark. Other models utilizing transformers consist of OpenAI-ChatGPT [71]. Typically, no one strategy consistently attains optimal predictive performance across every collection of data [72]. Consequently, regardless of the selected strategy, the model must be refined according to the particular dataset. Araci [73] observed that financial texts feature a specialized vocabulary, marked by unique terminology and a tendency toward ambiguous expressions instead of clearly defined positive or negative terms, which makes models trained on general corpora ineffective for this context. Consequently, he optimized BERT for financial data (FinBERT) and demonstrated its superiority over leading machine learning techniques for financial sentiment analysis datasets. The provided model was then applied in numerous studies and merged with other models, ultimately producing favorable results. For instance, Hiew et al [74] used a well-known pre-trained BERT model created by Google to analyze a text-based sentiment indicator, specifically for three individual stocks that are active in the Hong Kong market and have a lot of discussion on Weibo.com. They showed significant improvement in using BERT for financial sentiment analysis compared to existing models. Within the context of sentiment analysis, Wu et al [75] conducted a study examining the impact of deep learning techniques, with a particular focus on BERT models. The performance of LLM and FinBERT on news articles and company announcements was compared by Shen and Zhang [76] with respect to their applicability to FSA. Their study highlighted the advantages of quick engineering utilizing zero-shot and multi-shot methodologies to enhance sentiment categorization accuracy. To identify sentiment in financial documents, Jun Gu [77] and associates developed FinBERT, a pre-trained natural language processing model. Subsequently, this model was improved by integrating a LSTM architecture, and this novel model was named FinBERT-LSTM. The model employs news categories pertaining to the hierarchical structure of the stock market, namely those connected to the market, industry, and individual stocks, in conjunction with the prior week’s stock price status to generate predictions. Table 2 presents a summary of the literature review on sentiment analysis, organized by author, publication date, methodology used, and the data applied. Figure 1 provides an overview of the model categorizations.

**Table 2:** A summary of the research done in the area of sentiment analysis.

No.	Author	Year	Method	Data	Reference
1	Nasukawa and Yi	2003	Hybrid (Lexicon, NLP and Markov-model-based)	Web pages and news articles	[78]
2	Tetlock	2007	GI	Wall Street Journal	[79]
3	Schmeling	2009	Sentiment proxy	Monthly measure of consumer confidence	[80]
4	Joseph et al.	2011	Based on prior research on investor sentiment	Online ticker searches	[81]
5	Bollen et al.	2011	OpinionFinder (OF)Lexicon	Tweets	[82]
6	Preis et al.	2013	Google search volumes about financial markets	Google query volumes	[83]
7	Malo et al.	2014	LPS model	A collection of phrases/sentences sampled from financial news texts and company press releases	[84]
8	Guo et al.	2017	Thermal Optimal Path (TOP)	Site of China stock market called Xueqiu	[85]
9	Vaswani et al.	2017	Self-Attention	WMT and BlEU	[68]
10	Yang et al.	2018	Aspect-based sentiment analysis (ABSA)	Value Investors Club (VIC),FiQA	[86]
11	Devlin et al.	2019	Bert	GLUE,MultiNLI and SQuAD Dataset	[13]
12	Araci	2019	FinBERT	TRC2 and FiQA	[17]
13	Ko and Chang	2021	LSTM and BERT	News data and PPT	[87]
14	Leow et al.	2021	VADER and FinBERT	Tweets	[65]
15	Hiew et al.	2022	Bert	Weibo.com	[75]
16	Colasanto et al.	2022	FinBERT	Newspaper Articles	[88]
17	Fatouros et al.	2023	ChatGPT	News from Forex Live and FXstreet	[72]
18	Guo et al.	2024	FinBERT-LSTM	Benzinga news articles	[85]
19	Luo and Gong	2024	Llama2-7B	News Title	[89]
20	Shen and Zhang	2024	FinBERT	News and Reports	[77]
21	Wu et al.	2024	DistilBERT	SST2	[76]

In the field of sentiment analysis, the choice of pre-training data plays a crucial role in model performance. FinBERT is a specialized pre-trained language model developed specifically for sentiment analysis in the financial domain, having been trained on a large corpus of financial texts. While previous studies have predominantly relied on news articles, social media platforms (such as X), financial websites, and corporate statements as input sources, this study takes a different approach by utilizing companies’ quarterly reports to forecast future stock price movements using the FinBERT model. This choice is motivated by the fact that financial reports offer comprehensive and reliable insights into a company’s operations and future outlook.

### 2.3. Stock market prediction

In the last few years, the continuous development of big data technology and artificial intelligence (AI) has prompted an increasing number of researchers to use AI as the basis for their research methods, demonstrating that AI-based algorithms more effectively address the challenges of nonlinear and non-stationary features compared to traditional statistical models [90]. Notable methods include Support Vector Machine (SVM) [91], Principal Component Analysis (PCA) [92], Random Forest (RF) [93]and Genetic Algorithm (GA) [42], and Artificial Neural Network (ANN) [26,94]. A substantial quantity of review articles exists on stock price forecasting and prediction, thoroughly investigating the application of machine learning techniques in stock market forecasting.

Stock market prediction constitutes a regression application because of the continuous nature of stock prices [95]. Di Persio and Honchar [96] employed RNN for predicting Google stock prices. RNN, LSTM, and GRU are the three most effective neural networks for processing sequential data. RNN is utilized for analyzing historical data. LSTM and GRU circumvent the vanishing gradient problem through the utilization of forget, reset, and update gates. The GRU is determined to be quicker because to its utilization of reset and update gates. Future stock market prices can be predicted attributed to significant advancements in machine learning algorithms, particularly deep learning algorithms that are particularly good at identifying intricate linkages and patterns in financial time series data.

Because it can model long-term dependencies, LSTM, a kind of RNN, has garnered a lot of interest among these algorithms [97]. The following are some other studies that have been done on that subject matter. In a study, Bao et al [98] introduced an innovative deep learning architecture that integrates wavelet transformations (WT), stacked autoencoders (SAE), and LSTM for forecasting stock prices. SAEs were utilized for the inaugural time in forecasting stock prices based on hierarchically derived deep characteristics. Gulmes [99] has evaluated multiple algorithms, including LSTM1D, LSTM2D, LSTM3D, ANN, LSTM-GA, and LSTM optimized with ARO (LSTM-ARO), using the DJIA stock dataset from 2018 to 2023 in a study. Pang et al [100] introduced a deep long short-term memory (LSTM) neural network with an embedded layer and a long-term short-term memory (LSTM) neural network utilizing an auto encoder for stock market prediction. Gill et al [101] have examined the efficacy of advanced deep learning in a study. The research additionally introduces models for short-term trend prediction utilizing daily and hourly closing prices of the S&P 500 index and the Brazilian ETF EWZ. Liu et al. [102] developed a recurrent convolutional neural kernel (RCNK) model, which learns complementing characteristics from different data sources, primarily historical price data and text data from message boards, to predict stock fluctuations. Qiu et al [103] conducted an experiment using different wavelet functions and used SNR and RMSE criteria to determine the most effective wavelet for reducing stock price noise. Table 3 provides an overview of the literature review, summarizing key studies, methodologies, and findings related to the topic. It includes details such as the authors, publication dates, approaches used, and datasets analyzed, offering a comprehensive snapshot of the existing research in the field. Based on the findings from the literature analysis about the LSTM model (as indicated in Table 2) and its outstanding outcomes, this study will employ this model to forecast the expected returns.

**Table 3:** A summary of the research done in the area of stock market prediction.

No.	Author	Year	Method	Data	Reference
1	Dempser and Leemans	2006	Reinforcement Learning	FX data sets	[104]
2	Li et al.	2007	Reinforcement Learning Schemes	S&P 500 index NASDAQ	[105]
3	Kara et al.	2011	A hybrid model (ANN-SVM)	Istanbul Stock Exchange	[106]
4	Huang	2012	A hybrid model (Genetic Algorithms (GA) and Support Vector Regression (SVR))	Taiwan Stock Exchange	[107]
5	Kao et al.	2013	A hybrid model (NLICA-SVR)	Asian stock markets-China and Japan (Shanghai and Nikkei)	[108]
6	Rather et al.	2015	A Hybrid Prediction Model with RNN	Bombay Stock Exchange	[109]
7	Kraus and Feuerriege	2017	Deep Neural Networks and Transfer Learning	Different corpus with a length of 139.1 million words	[110]
8	Tsantekidis et al.	2017	Convolutional Neural Networks (CNN)	Nasdaq	[111]
9	Chong et al	2017	Deep Feature Learning-Based Stock Market Prediction Model	Korean stock market	[96]
10	Bao et al.	2017	Hybrid Approach (wavelet transforms (WT), SAEs and LSTM)	Six market indices and their corresponding index futures	[100]
11	Ta et al.	2018	Linear Regression (LR) and Support Vector Regression (SVR)	S&P 500 ETF-SPY	[112]
12	Fischer and Krauss	2018	Deep Learning with Long Short-Term Memory Networks	S&P 500 index constituents from Thomson Reuters	[113]
13	Baek and Kim	2018	ModAugNet(an overfitting prevention LSTM module and a prediction LSTM module)	S&P500 and Korea Composite Stock Price Index 200 (KOSPI200)	[114]
14	Liu	2019	Deep Learning Long Short-Term Memory Recurrent Neural Networks	S&P 500	[115]
15	Ma et al.	2020	DNNs, DMLP, LSTM and CNN	China securities 100 index	[21]
16	Zhang et al.	2020	CEEMD-PCA-LSTM	Shanghai Composite Index	[116]
17	Sun et al.	2020	Ensemble Deep Learning Approach called LSTM-B	US dollars (USD) against other four major currencies, such as GBP, JPY, EUR and CNY	[117]
18	Lei et al.	2020	TFJ-DRL (Deep learning and Reinforcement learning)	U.S. stocks(S&P500) from yahoo finance	[118]
19	Wang et al.	2020	Mixed Method (LSTM and MV)	UK Stock Exchange	[60]
20	Chen et al.	2021	A Hybrid Model Based on Machine Learning (XGBoost and IFA)	Shanghai Stock Exchange	[119]
21	Ma et al.	2021	A hybrid model (random forest (RF) and support vector regression (SVR))	China securities 100 index	[120]
22	M et al.	2022	Holt–Winters algorithm and recurrent neural network LSTM	Fifteen companies from different sectors	[121]
23	Aldhyani and Alzahrani	2022	Long Short-Term Memory (LSTM) and a hybrid of a Convolutional Neural Network (CNN-LSTM)	Tesla, Inc. and Apple, Inc.	[122]
24	Mukherjee et al.	2023	Deep Learning Algorithms	National Stock Exchange (NSE) stock market dataset, specifically the NIFTY price index	[123]
25	Sarma et al.	2023	Deep Learning Algorithms (LSTM, CNN)	-	[124]
26	Chavha et al.	2024	A Comparative Study of LSTM, RNN, and GRU Models	GOOG, AAPL, MSFT, GME	[125]
27	Gautam et al.	2024	Linear Forecast, Naive Forecast, LSTM and ARIMA	NABIL dataset	[126]
28	Behura et al.	2024	Multi-Layered Sequential LSTM	National Stock Exchange (NSE)	[127]
29	Furizal et al.	2024	Hybrid LSTM	S&P500 dataset	[128]

Various extensions of Markowitz’s MV model improve modern portfolio theory and provide researchers with additional perspectives for research. These developments highlight the importance of improving the MV model in portfolio management. In general, both short-term and long-term investment methods can be used to purchase stocks listed on the stock market. Long-term investment involves holding stocks for a long time, while short-term investment involves buying and selling stocks in shorter time frames, and investors aim to make a profit within a few days or weeks [102]. Traders use several trading tactics, such as short-term, long-term, and intermediate-term [103]. However, no previous research has simultaneously examined the three objective functions of risk, return, and utility (the trend prediction score obtained from sentiment analysis of text data) for a portfolio. To achieve quarterly long-term investment, we incorporate a new objective function into the original model in this study to maximize utility. This utility function takes into account the impact of environmental data (quarterly reports) in the portfolio formation model. The presented model was finally implemented on real data of index DJIA companies and then solved using the WGT method and the results were presented. The methodology will be examined in the next section.

## 3. Methodology

In this paper, we propose a three-objective model for portfolio optimization. For the return data (expected return), we use the LSTM method. The goal of financial market forecasting is not to validate a model, but to transform its successful results into actionable insights that can provide investors with more practical and useful recommendations in real market conditions [36]. This is something that many researchers do not consistently consider. When it comes to the investment decision-making process, having high-quality asset inputs will greatly help in forming the best possible portfolio. Since the MV technique is fundamental for portfolio management, we will continue to rely on this classic model for our approach. Furthermore, we combine the general trend forecast of future price movements using quarterly reports of companies with the MV model to obtain the best possible combination. Furthermore, we will use the LSTM model to calculate the expected return of companies. The model presented in this article can be summarized as follows.

- Step 1: This step focuses on sentiment analysis, where we analyze text data from companies’ quarterly reports to gauge market sentiment. By calculating sentiment scores, we aim to predict future market trends and assess the potential impact of public sentiment on asset performance.
- Step 2: This step uses historical stock data to predict future price movements. We forecast stock prices for the second quarter using the LSTM algorithm, a powerful deep learning technique known for its effectiveness in handling sequential data. This approach allows us to capture complex patterns and dependencies in the historical data, increasing the accuracy of our price predictions.
- Step 3: In the final step, we integrate insights from sentiment analysis and price forecasting to determine the optimal asset portfolio. This is achieved through a modified mean-variance model, which balances risk and return by considering the expected returns from LSTM forecasts and sentiment-based market outlook. By modifying the traditional mean-variance framework, our model provides a modified and adaptive strategy for portfolio optimization, tailored to the current market environment.

**Fig 2:**
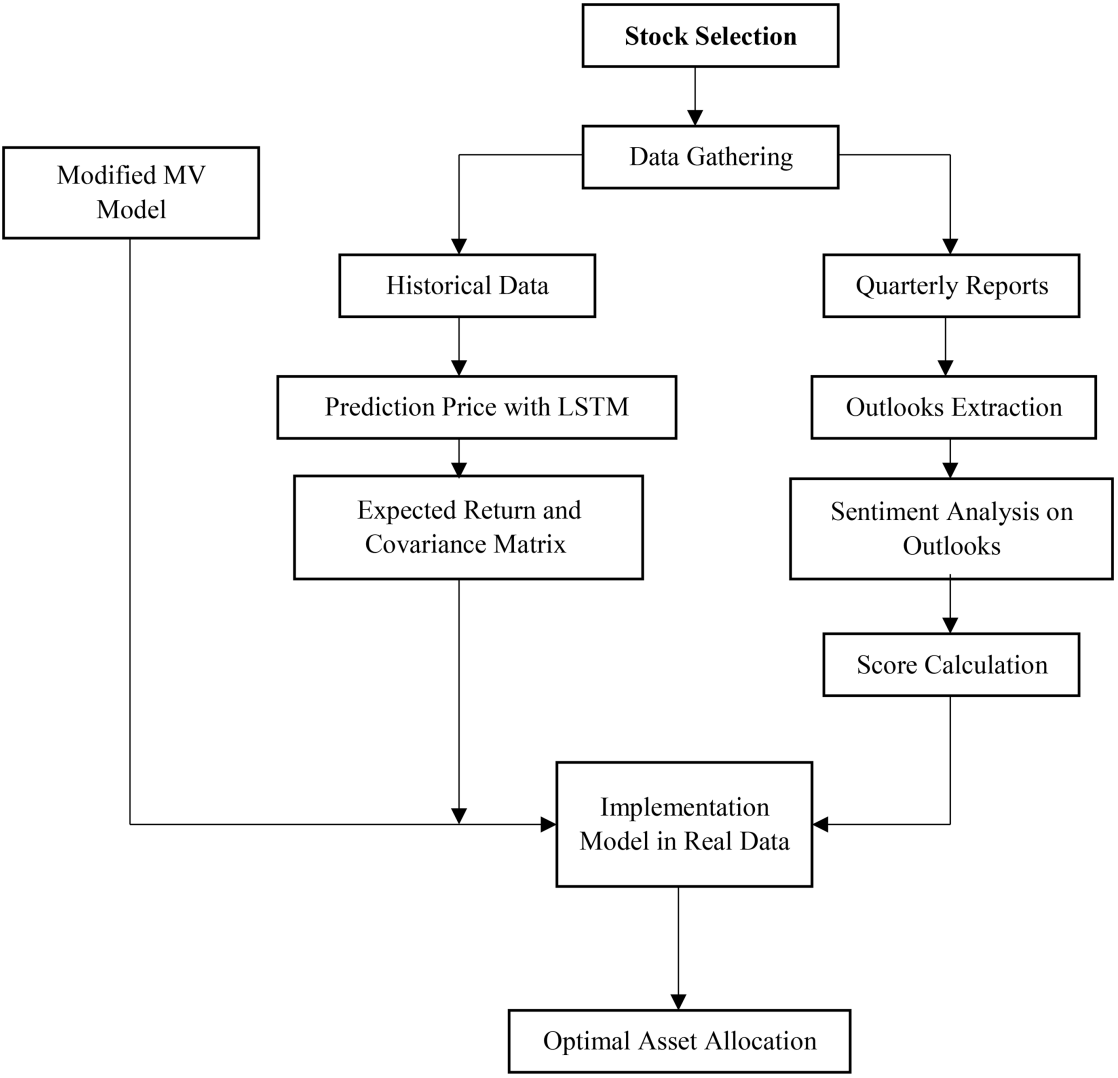
A visual outline summarizing all the steps involved in the proposed model.

Together, these three steps form a robust framework that combines qualitative sentiment analysis, quantitative price forecasting, and advanced portfolio optimization techniques. The goal of this integrated approach is to provide investors with a data-driven adaptive strategy to maximize returns while managing risk in dynamic financial markets. The proposed methodology is illustrated visually in Fig 2, which shows the step-by-step workflow of our approach. This flowchart provides a clear and structured overview of the entire process and guides readers through each step of the analysis. As illustrated in Fig 2, the workflow begins with the selection of the proposed stocks and proceeds with the collection of initial data, followed by sentiment analysis. The flowchart demonstrates how sentiment scores are computed and incorporated into the framework. Next, it transitions to the application of the LSTM algorithm for stock price prediction, emphasizing the utilization of historical data to generate forecasts. The process concludes with the optimization phase, where a modified mean-variance model is employed to determine the optimal asset mix, leveraging the combined insights derived from sentiment analysis and price predictions. In the following sections, we provide concise overviews and definitions of the FinBERT, LSTM, and mean-variance models, outlining their key characteristics and roles within the proposed framework.

### 3.1. *FinBERT model*

**Fig 3:**
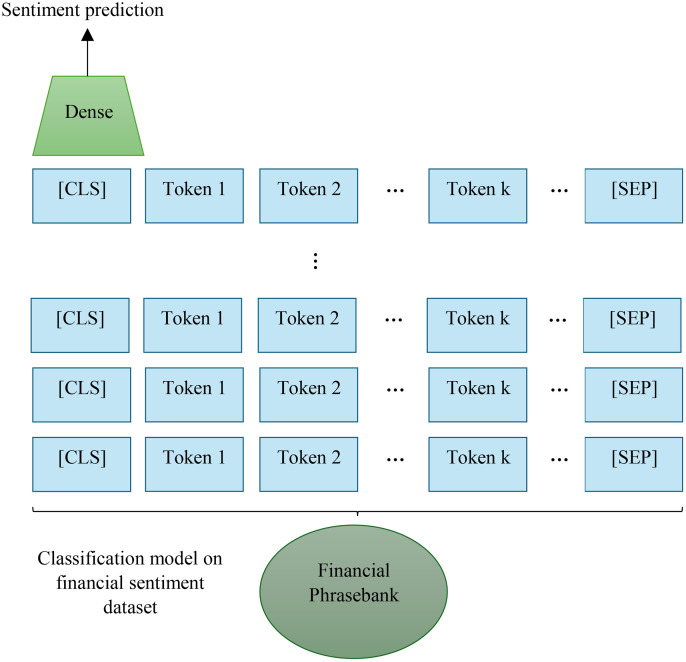
FinBERT architecture following Araci [17].

Analyzing financial sentiment is challenging due to the specialized vocabulary and the limited availability of labeled data in this domain [17]. This study utilizes the pre-trained FinBERT model to conduct sentiment analysis and predict future fluctuations in stock prices. FinBERT is specifically designed for financial text analysis, offering improved accuracy over general-purpose language models when applied to financial content. In this section, we elaborate on the model’s architecture, theoretical foundations, and its advantages for sentiment classification within the financial domain. The FinBERT model is based on the widely adopted BERT framework, but it is uniquely fine-tuned on large-scale financial corpora. This enables the model to better understand the nuanced language and context typical of financial documents. Through domain-specific training and task-oriented fine-tuning, FinBERT achieves a high level of precision in detecting sentiment signals from textual data. The detailed architecture of the FinBERT model is illustrated in Fig 3, highlighting the components tailored for financial sentiment analysis.

The primary function of the input layer is to accept financial text and transform it into numerical vectors suitable for model processing, utilizing techniques such as word segmentation and word embedding. The BERT encoder employs a multilayer transformer architecture that effectively collects contextual information from the text via the attention mechanism, generating a corresponding vector representation of the text. A sentiment classification layer is subsequently incorporated atop the BERT encoder, utilizing the encoder’s output as input to execute sentiment classification over a fully linked network. The sentiment categorization layer comprises several neurons, each representing a distinct sentiment category. The training procedure of the FinBERT Sentiment model is primarily segmented into two phases: pre-training and fine-tuning.

- Pre-training: The pre-training FinBERT model comprises two primary phases:
  1. Pre-training: the original BERT model is trained on extensive, general datasets (such as BookCorpus and Wikipedia) to comprehend general language and sentence structure. This phase is non-specialized and emphasizes general language understanding.
  2. Further Pre-training: the model undergoes additional training on financial datasets, including news articles, financial reports, and corporate announcements. The objective is to acquire financial terminology (e.g., “stocks,” “bonds,” “profit,” “loss”) and boost expertise in financial NLP tasks, such as sentiment analysis and named entity recognition, to achieve better accuracy and efficiency.
- Fine-tuning: during the fine-tuning process, the model is trained using dataset specific to finance. The goal is for the model to adjust its weights to process financial language more effectively while retaining the broad language understanding gained in the pre-training phase. In this phase, the model is trained on labeled data using techniques such as backpropagation and gradient descent. A reduced learning rate is typically used during fine-tuning to avoid disturbing the general language knowledge already acquired by BERT.

The FinBERT model generally has the following advantages over other models:

- *Domain-Specific Training:* FinBERT is trained on financial texts, giving it a deeper understanding of financial terminology and context.
- *Improved Sentiment Analysis:* It provides more accurate sentiment classification for financial content like earnings reports or market news.
- *Better Context Understanding:* FinBERT handles ambiguous financial terms (e.g., “bullish,” “liability”) more accurately than general models.
- *Higher Accuracy in Financial Tasks:* It outperforms general models in tasks such as financial entity recognition and document classification.
- *Reduced Noise in Predictions:* Because it’s specialized, FinBERT generates fewer irrelevant or incorrect outputs when analyzing financial texts.
- *Time and Resource Efficiency:* Using a pre-trained domain-specific model like FinBERT reduces the need for extensive retraining on financial datasets.

### 3.2. *LSTM networks*

**Fig 4:**
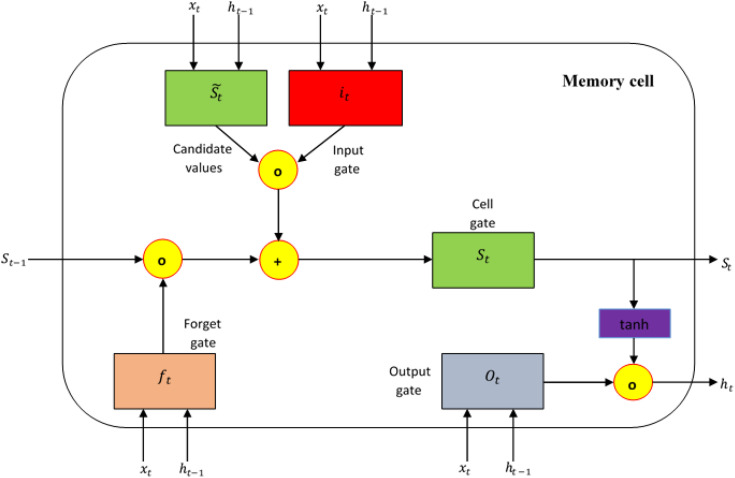
The architecture of the LSTM memory cell, as outlined by Fischer and Krauss [29].

LSTM networks were first introduced by Hochreiter and Schmidhuber [24] as a technique for capturing and learning sequential patterns. LSTM networks are a specialized form RNNs that are capable of retaining information over extended periods, making them more effective than traditional RNNs in handling long-term dependencies [62]. Graves and Schmidhuber [121] provide evidence that LSTM networks are capable of overcoming the inherent issues that were previously present and memorizing temporal patterns over an extended length of time. Consequently, in our study, we will make use of this model to forecast the prices of stocks in the future. LSTM networks are composed of an input layer, several hidden layers, and an output layer. What distinguishes LSTMs from other types of networks is the inclusion of memory cells within the hidden layers. Fig 4 shows the architecture of an LSTM memory cell, which plays a crucial role in storing and managing information over time. To effectively model time-series data such as stock prices, the LSTM model is configured with the following components:we use quarterly repor

1. *Input Layer:* The input layer takes in a sequence of historical financial data, such as the opening price, closing price, high, low, and trading volume. This input is typically structured into time windows (e.g., 60-time steps) to give the model temporal context. Each input vector at a time step can include multiple features.
2. *LSTM* *Layers:* The core of the model is the LSTM layer (or layers), which consists of memory blocks called cells.Each cell maintains an internal state, controlled by three gates:
  - **Forget Gate (f_t_):** Decides what information to discard from the cell state.
  - **Input Gate (i_t_):** Determines which new information to store in the cell state.
  - **Output Gate (o_t_):** Controls how much of the internal state to pass to the output.

The input and hidden states are represented by $x_{t}$ and $h_{t}$ at time t. respectively, $s_{t}$ is adjusting its cell state. The input gate controls which data should be stored in the memory cell, the output gate governs what information is extracted from the memory cell, and the forget gate determines which data should be discarded. The mathematical formulation of an LSTM cell is as follows:


$$\begin{array}{c} f_{t}=sigmoid\left(W_{f,x}x_{t}+W_{f,h}h_{t-\text{1}}+b_{f}\right) \end{array}$$ (1)

$$\begin{array}{c} \tilde{s_{t}}=sigmoid\left(W_{\tilde{s},x}x_{t}+W_{\tilde{s},h}h_{t-\text{1}}+b_{\tilde{s_{t}}}\right) \end{array}$$ (2)

$$\begin{array}{c} i_{t}=sigmoid\left(W_{i,x}x_{t}+W_{i,h}h_{t-\text{1}}+b_{i}\right) \end{array}$$ (3)

$$\begin{array}{c} s_{t}=f_{t}*s_{t-\text{1}}+i_{t}*\tilde{s_{t}} \end{array}$$ (4)

$$\begin{array}{c} o_{t}=sigmoid\left(W_{o,x}x_{t}+W_{o,h}h_{t-\text{1}}+b_{o}\right)\; \end{array}$$ (5)

$$\begin{array}{c} h_{t}=o_{t}*\text{tanh}(s_{t}) \end{array}$$ (6)

Where $W_{f,x}$,$\;W_{f,h}$,$\;W_{\tilde{s},x}$,$\;W_{\tilde{s},h}$,$\;W_{i,x}$,$\;W_{i,h}$,$\;W_{o,x}$and $W_{o,h}$ are weight matrices, $b_{f}$, $b_{\tilde{s}}$, $b_{i}$, and $b_{o}$ are the bias vectors associated with each of the respective gates. The bias vectors are incorporated to enhance the model’s adaptability to the data. The bias vectors, $b_{\tilde{s}}$, $b_{i}$, and $b_{o}$ are initialized to zero, whereas the bias $b_{f}$ the forget gate in the LSTM is initialized to 1.0. The symbol * represents element-wise multiplication. These operations allow the model to regulate information flow dynamically and effectively manage both short-term and long-term dependencies.

1. *Stacked LSTM Architecture:* In many advanced configurations, multiple LSTM layers are stacked to increase the model’s representational power. The output of each LSTM layer is passed to the next layer, enabling deeper abstraction of sequential features.
2. *Dropout Regularization:* Dropout layers are inserted between LSTM layers to prevent overfitting by randomly deactivating a fraction of the neurons during training. This improves generalization, especially when the dataset is small or noisy.
3. *Dense (Fully Connected) Layer:* The final dense layer maps the high-level sequence representations to a single output value (e.g., predicted stock price). For regression tasks, the activation function is typically linear.
4. *Compilation and Training:* The model is compiled using a loss function such as Mean Squared Error (MSE), which is appropriate for continuous output variables. The optimizer is often Adam, which adapts learning rates and accelerates convergence. Training is conducted over multiple epochs, with early stopping or validation monitoring to prevent overfitting.

The architecture of the LSTM network is particularly advantageous for modeling financial time series data, where both short-term fluctuations and long-range temporal dependencies play a critical role in shaping future trends. In the context of stock market forecasting, price movements are rarely independent of historical behavior; rather, they are influenced by recurring patterns, investor sentiment, and macroeconomic events that unfold over varying timescales. LSTM networks are inherently designed to capture such temporal structures by maintaining a memory of past states while dynamically adjusting their focus to recent changes. This ability allows them to outperform traditional machine learning models, which typically rely on fixed-size input vectors and handcrafted features. Unlike methods such as linear regression or support vector machines that require domain-specific feature engineering to capture time dependencies, LSTM models automatically learn relevant temporal representations directly from raw sequential data. This makes them not only more adaptable to the complex dynamics of financial markets but also more scalable for real-world forecasting applications where patterns may shift over time. For more information, please refer to the article by Yu et al [124].

### 3.3. *Mean-variance (MV) model*

MV model was established by Markowitz [2] in order to address the process of choosing the optimal portfolio problem. This model is the foundation upon which MPT is built. Both the return on investment and the risk associated with it are defined in this approach by the expected return and the variance, respectively. According to Santos and Tessari [125], The key factor in portfolio selection for investors is identifying the portfolio that best suits their preferences, considering the associated risk level and expected returns. Consequently, rational investors tend to prefer portfolios with lower risk and stable expected returns, or those with higher expected returns but consistent risk levels. To address this issue, a set of optimal alternatives is created, referred to as the efficient investment frontier. The model is defined by the following set of equations:


$$\begin{array}{c} Min\;\sum_{i=\text{1}}^{n}\sum_{j=\text{1}}^{n}w_{i}w_{j}\delta_{ij} \end{array}$$ (7)

$$\begin{array}{c} Max\;\sum_{i=\text{1}}^{n}w_{i}\mu_{i}\; \end{array}$$ (8)

Subject to:


$$\begin{array}{c} \sum_{i=\text{1}}^{n}w_{i}=\text{1} \end{array}$$ (9)

$$\begin{array}{c} \text{0}\leq w_{i}\leq \text{1}\;\forall \;i=\text{1},\ldots ,n \end{array}$$ (10)

The objective function aimed at minimizing risk is expressed in Equation 7, while the expected return of the portfolio comprising the chosen stocks is determined using Equation 8., and $w_{i}$ and $w_{j}$ denote the percentage of the total investment that is allocated to assets 𝑖 and j, respectively. The $\delta_{ij}$ is used to specify the covariance between assets *i* and asset *j* and $\mu_{i}$ describes the expected return on *ith* asset. Equation 9 specifies that the proportion of budget distributed between different stocks must sum to 1. Equation 10 states that the investment % per share can never be negative. The following section provides a detailed explanation of the underlying mathematical modeling of the proposed approach, including the formulation of key equations, the assumptions made, and the rationale behind the chosen solution technique.

## 4. Model formulation

In this section, a comprehensive discussion of the final mathematical model is presented, its structure is examined in detail and its key components are highlighted. Then, the proposed solution methodology is thoroughly reviewed, its main features are described. Finally, the revised and finalized model is introduced, which integrates both structural modifications and solution methodology to effectively address the underlying problem. This step-by-step description is intended to provide a clear understanding of the model development and the rationale behind the approach adopted.

### 4.1. *A modification of the fundamental model*

By incorporating a third objective function into the basic model that was discussed in Section 3.4, we further develop the model in this paper. A representation of the extended mathematical model can be shown following.


$$\begin{array}{c} Min\;\sum_{i=\text{1}}^{n}\sum_{j=\text{1}}^{n}w_{i}w_{j}\delta_{ij} \end{array}$$ (11)

$$\begin{array}{c} Max\;\sum_{i=\text{1}}^{n}w_{i}\mu_{i} \end{array}$$ (12)

$$\begin{array}{c} Max\;\sum_{i=\text{1}}^{n}\gamma_{i}w_{i}\; \end{array}$$ (13)

Subject to:


$$\begin{array}{c} \sum_{i=\text{1}}^{n}w_{i}=\text{1} \end{array}$$ (14)

$$\begin{array}{c} \text{0}\leq w_{i}\leq \text{1}\;\forall \;i=\text{1},\ldots ,n \end{array}$$ (15)

Additionally, $\gamma_{i}$ represents the degree of bullish trend for the *ith* asset in the upcoming quarter, as determined by the FinBERT model. Section 3.5 provides clarification on additional indicators and marks. In the next section, the proposed solution method is discussed, and finally, the final modified model is presented.

### 4.2. *Proposed approach for solving multi-objective models*

In this research, we apply the Goal Programming (GP) approach to tackle multi-objective optimization problems within the proposed model. GP was first developed by Charnes et al. [124] and Charnes and Cooper [125]. Romero [126], Jones and Tamiz [127], and Ignizio [128] all contributed to its continued development. Romero states that the achievement function, which gauges the extent to which the undesirable deviational variables of the model’s objectives have been minimized, is a crucial component of a GP model. This section presents a concise overview of the GP methods and normalization techniques employed in this paper. For a detailed overview of GP, including its various types and the normalization techniques employed, the reader is directed to the work of Jones and Tamiz [127].

#### 4.2.1. *The theory of the Weighted Goal Programming (WGP) approach.*

WGP is generally considered a deterministic approach that provides effective solutions for addressing multi-objective challenges in technological and economic contexts, operating within the scope of multi-criteria decision-making analysis [129]. WGP is a form of goal programming where each objective is allocated a distinct weight to signify its relative significance in the decision-making procedure. The purpose is to reduce discrepancies from the target objectives by taking into account varying priorities or the relative significance of each goal. The general WGP model:


$$\begin{array}{c} Min\;\sum_{i=\text{1}}^{m}\frac{\alpha_{i}n_{i}}{k_{i}}+\frac{\beta_{i}p_{i}}{k_{i}} \end{array}$$ (16)

Subject to:


$$\begin{array}{c} f_{i}(x)+n_{i}-p_{i}=b_{i}\;i=\text{1},\text{2},\ldots ,m \end{array}$$ (17)

$$\begin{array}{c} x\in C_{s} \end{array}$$ (18)

$$\begin{array}{c} x\geq \text{0}\;,\;n_{i,\;},p_{i}\geq \text{0}\;i=\text{1},\text{2},\ldots ,m\; \end{array}$$ (19)

Note that the normalization constant for deviant variables is $k_{i}$, the negative deviant variable *i* is $n_{i}$, the positive deviant variable *i* is $p_{i}$, the weight assigned to positive factors deviant variable *i* is $\beta_{i}$, and the weighting factor for the negative deviant variable is $\alpha_{i}$. $C_{s}$ Is an optional set of hard limitations, $b_{i}$ is the ith target value, $f_{i}(x)$ is the *ith* objective function, and *x* is the vector containing the decision variables. The WGP facilitates direct trade-offs among all unwanted deviational characteristics by including them into a weighted, normalized singular achievement function that is minimized. Each goal, $f_{i}(x)$, is assigned a target value, $b_{i}$, that must be attained. The positive deviational variable, $p_{i}$, indicates overachievement, whereas the negative deviational variable, $n_{i}$, indicates how much the aim was underachieved. The achievement function is then used to minimize the unwanted deviation from the objective value. The decision maker’s preferences are reflected in the values of α and β. If attaining the profit aim of $b_{k}$k is deemed twice as significant as meeting the capacity target of $b_{l}$, then the associated deviational variables are assigned weights of $\alpha_{k}$ = 2 and $\beta_{l}$ = 1 in the achievement function. The weights in GP plays two roles: (a) they normalize the unit and measurement scales, and (b) they value the decision maker’s preferences. The preferences of the decision maker can be incorporated into Goal Programming (GP) models by:

- Weights and penalties for the undesirable deviational variables;
- Choosing the target values for each objective.

A normalization approach is used in GP to quantify deviations from each aim using a consistent unit of measurement. Prominent normalizing approaches include Euclidean, percentage, and zero-one methods. Euclidean normalization is especially advantageous when the goal values are near zero. Thus, the Euclidean mean of the objective function’s technical coefficients is the normalization constant. $k_{i}$. According to Jones and Tamiz [127], Euclidean normalization is a computationally robust technique that is applicable to all goals, even those with minimal or zero target values, and does not need optimization or intricate computations to get the normalization constants. For the following goal *i*, the normalization constant, denoted by. $k_{i}$, is as follows:


$$\begin{array}{c} \sum_{j=\text{1}}^{n}a_{ij}x_{j}+n_{i}-p_{i}=b_{i} \end{array}$$ (20)

Is calculated as:


$$\begin{array}{c} k_{i}=\sqrt{\overset{\text{2}}{\underset{i\text{1}}{a}}+\overset{\text{2}}{\underset{i\text{2}}{a}}+\ldots +\overset{\text{2}}{\underset{in}{a}}} \end{array}$$ (21)

#### 4.2.2. Final model.

Building upon the explanations provided in the previous sections, the proposed model has been systematically reformulated to incorporate the identified modifications and improvements. This revised formulation reflects the underlying assumptions, structural adjustments, and methodological choices made during the development process. The updated model is presented as follows to clearly illustrate these enhancements and their role in addressing the problem more effectively.


$$\begin{array}{c} Min\;\frac{\alpha_{re}}{k_{re}}n_{re}+\frac{\alpha_{dt}}{k_{dt}}n_{dt}+\frac{\beta_{ri}}{k_{ri}}p_{ri} \end{array}$$ (22)

Subject to:


$$\begin{array}{c} \sum_{i=\text{1}}^{n}\sum_{j=\text{1}}^{n}w_{i}w_{j}\delta_{ij}-p_{ri}=b_{\text{1}}\; \end{array}$$ (23)

$$\begin{array}{c} \sum_{i=\text{1}}^{n}w_{i}\mu_{i}+n_{re}=b_{\text{2}} \end{array}$$ (24)

$$\begin{array}{c} \sum_{i=\text{1}}^{n}\gamma_{i}w_{i}+n_{dt}=b_{\text{3}}\; \end{array}$$ (25)

$$\begin{array}{c} \sum_{i=\text{1}}^{n}w_{i}=\text{1} \end{array}$$ (26)

$$\begin{array}{c} \text{0}\leq w_{i}\leq \text{1}\;\forall \;i=\text{1},\ldots ,n\; \end{array}$$ (27)

Where $n_{re}$,$\;n_{dt}$, $n_{ri}$ represents the negative deviation variables and $p_{re}$,$\;p_{dt}$,$\;p_{ri}\;$represents the positive deviation variables. $b_{\text{1}}$,$\;b_{\text{2}}$ and $b_{\text{3}}$ represent the ideal values of the objective functions obtained by solving the single-objective model while accounting for additional constraints. In the next section, the proposed model is implemented on real data of the companies that make up the Dow Jones Index and its results are presented.

## 5. Data and experiments

This section provides a detailed and structured overview of the data collection and model implementation process. It is divided into four distinct sections to ensure clarity and accuracy. First, the basic steps involved in collecting the required data are discussed, emphasizing its importance in ensuring the accuracy and reliability of the analysis. Next, the application of the FinBERT model to reporting quarterly data is examined, focusing on its ability to determine the direction of the trend, which serves as a critical input for the subsequent steps. Following this, the results produced by the LSTM model are examined, which are used to calculate the regression values and the covariance matrix – key components of the mathematical model. Finally, the results obtained from solving the proposed model are presented, highlighting the optimal asset allocation strategies identified.

### 5.1. Data

One of the primary challenges in financial forecasting lies in identifying meaningful relationships between historical and future events within time-series data [37]. In this study, we rely on two main categories of data: numerical historical data and unstructured textual data. The focus of our case study is on the companies listed in the DJIA, which consists of 30 major publicly traded firms. Accordingly, both historical and textual information have been collected for each of these 30 companies to support our analysis.

- Textual data: For the purpose of gathering textual data, we have utilized the quarterly reports that were released by firms (those that are included in the DJIA) during the first quarter of each year 2024. The reports in question were obtained from the Yahoo Finance website, and the Sider AI was utilized in order to extract the company’s outlook for the upcoming quarter. As an illustration of the extracted outlook, Table 4 provides an example.
- *Historical data:* Historical data is needed as input for the LSTM model. To do this, we have gathered historical data (daily closing prices) for the firms comprising the DJIA utilizing the yfinance library. Yfinance is an open-source library for acquiring and retrieving financial data, including business calendars, historical data, etc. In the Python programming language. We will utilize historical data from 2015 through the conclusion of the first quarter of 2024 as input for the LSTM model.

**Table 4:** Sample of company quarterly report outlooks.

Symbol	Company	Date	Outlook
BA	The Boeing Company	02, may,2024	The outlook for The Boeing Company in the next quarter appears to be cautious, primarily due to ongoing challenges related to production and quality control, particularly with the 737 MAX aircraft. The company has recently faced significant operational disruptions, including the grounding of 737−9 aircraft following an emergency incident and subsequent FAA inspections. These issues have led to a slowdown in production rates and a reduction in aircraft deliveries, which are expected to adversely impact revenues and cash flows in the near term. In the first quarter of 2024, Boeing reported a net loss of 355million, with revenues decreasing to 355 million, with revenues decreasing to 16.57 billion from 17.92 billion in the same period the previous year. The loss from operations was 17.92 billion in the same period the previous year. The loss from operations was 86 million, an improvement from the prior year but still indicative of ongoing challenges. The company has also indicated that revenues will continue to be significantly impacted until the global supply chain stabilizes and labor issues are resolved. Looking ahead, Boeing’s management has emphasized the need to improve compliance with manufacturing quality control requirements and is working on a comprehensive action plan to address the issues identified by the FAA. The company expects that these operational challenges will persist until production rates can recover, which could further affect financial performance in the upcoming quarters. Overall, while there may be some positive developments in other segments, such as Defense, Space & Security and Global Services, the immediate outlook for the next quarter remains uncertain and heavily influenced by the ongoing issues with the 737 program and broader supply chain constraints.
GS	The Goldman Sachs Group, Inc.	03, may,2024	The future outlook for The Goldman Sachs Group, Inc. appears cautiously optimistic, but it is tempered by various macroeconomic uncertainties and potential risks. Here are the key points regarding the company’s outlook: **1. Economic Environment:** The global economy is experiencing growth, but concerns about inflation, geopolitical tensions (notably with China and conflicts in Ukraine and the Middle East), and the commercial real estate sector persist. These factors could impact market conditions and client activity levels **2. Financial Performance:** Goldman Sachs reported a significant increase in net earnings for the first quarter of 2024, with net revenues rising 16% compared to the same period in 2023. This growth was driven by higher revenues across all business segments, particularly in investment banking and asset management. **3. Investment Banking Activity:** While investment banking fees increased, there are indications that the backlog for future transactions has decreased, suggesting potential challenges ahead in maintaining high levels of activity in this area. **4. Asset & Wealth Management:** The firm aims to achieve pre-tax profitability in its Platform Solutions segment by the end of 2025. However, the outlook for this segment is contingent on economic conditions and consumer spending, which could be negatively impacted if economic conditions deteriorate. **5. Credit Loss Provisions:** The company has increased its provisions for credit losses, reflecting concerns about the credit quality of its portfolios, particularly in consumer lending. Future credit losses may vary based on economic conditions and the performance of borrowers. **6. Regulatory Environment:** Goldman Sachs operates in a heavily regulated environment, and potential changes in regulations could impact its capital requirements and operational flexibility. **7. Market Conditions:** The firm is closely monitoring key metrics related to market conditions, including interest rates and asset prices. A decline in market conditions could negatively affect its revenues and profitability.**8. Strategic Initiatives:** Goldman Sachs is pursuing various strategic initiatives, including the transition of its GM credit card program and potential sales of historical principal investments in its Asset & Wealth Management segment. The success of these initiatives will depend on market conditions and execution. In summary, while Goldman Sachs has shown strong financial performance recently, its future outlook is influenced by a complex interplay of economic conditions, regulatory changes, and market dynamics. The company remains focused on achieving its profitability targets while navigating these challenges.

The constituents of the DJIA comprise “UnitedHealth Group, Inc.” (UNH), “The Goldman Sachs Group, Inc.” (GS), “Microsoft Corporation” (MSFT), “The Home Depot, Inc.” (HD), “Caterpillar, Inc.” (CAT), “Amgen Inc.” (AMGN), “McDonald’s Corporation” (MCD), “Salesforce, Inc.” (CRM), “Visa, Inc.” (V), “American Express Company” (AXP), “The Travelers Companies, Inc.” (TRV), “Apple, Inc.” (AAPL), “International Business Machines Corporation” (IBM), “JPMorgan Chase & Co.” (JPM), “Honeywell International, Inc.” (HON), “Amazon.com, Inc.” (AMZN), “The Procter & Gamble Company” (PG), “Johnson & Johnson” (JNJ), “The Boeing Company” (BA), “Chevron Corporation” (CVX), “3M Company” (MMM), “Merck & Co., Inc.” (MRK), “The Walt Disney Company” (DIS), “NIKE, Inc.” (NKE), “Walmart, Inc.” (WMT), “The Coca-Cola Company” (KO), “Cisco System, Inc.” (CSCO), “Dow Inc.” (DOW), “Verizon Communication, Inc.” (VZ) and “Intel Corporation” (INTC).

In this study, the choice of DJIA data was driven by several factors:

1. **Availability of high-quality, continuous data:** DJIA companies provide comprehensive and reliable datasets, allowing us to rigorously evaluate the model.
2. **Focus on a specific market to control variability:** Concentrating on a single, well-defined index helps reduce complexity arising from structural differences across various markets.
3. **Time and resource constraints:** Access to a broader and more diverse dataset was limited during the research period.

### 5.2. Details *on usage of the FinBERT model*

**Fig 5:**
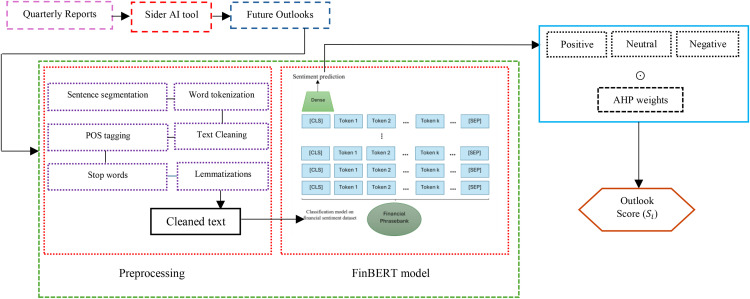
The process of extracting corporate Outlook scores.

As mentioned in Section 5.1, we use quarterly reports to predict future price outlooks. We collect companies’ outlooks for the second quarter after the first quarterly reports are received in 2024 and use this text data as input to the FinBERT model. Also, this paper uses the Python Transformer package and the *ProsusAI/finbert* pipeline for the model input. The model output findings are presented in Table 5. The process of extracting the score for company future outlooks is as follows: first, companies’ quarterly reports are extracted, and then insights and information from these reports are extracted using the Sider AI tool. two of the outlooks (future trends) extracted with the help of the Sider artificial intelligence tool are shown as examples in Table 4, and the complete data has been uploaded to GitHub and its link is as follows https://github.com/esipour93/DJIA_Textual_Data-1.git. The extracted outlooks are then fed to the FinBERT model and sentiment analysis is performed on them, and then the FinBERT model outputs are converted into $\gamma_{i}$ scores using the AHP method. This process is graphically shown in Fig 5.

**Table 5:** Demonstrating the results of the FinBERT model.

Stock	*Sentiment analysis*			$\gamma_{𝐢}$	Stock	*Sentiment analysis*			$\gamma_{𝐢}$
	positive	neutral	negative			positive	neutral	negative	
UNH	0.307	0.101	0.592	**0.188018**	AMZN	0.929	0.039	0.032	**0.660876**
GS	0.919	0.046	0.035	**0.654661**	PG	0.951	0.021	0.028	**0.672744**
MSFT	0.958	0.028	0.014	**0.680122**	JNJ	0.734	0.06	0.206	**0.513426**
HD	0.062	0.027	0.911	**−0.027117**	BA	0.013	0.017	0.971	**−0.068642**
CAT	0.017	0.026	0.957	**−0.062917**	CVX	0.822	0.071	0.108	**0.586129**
AMGN	0.903	0.049	0.048	**0.642922**	MMM	0.396	0.042	0.562	**0.241254**
MCD	0.837	0.152	0.011	**0.621233**	MRK	0.125	0.025	0.85	**0.02225**
CRM	0.797	0.066	0.138	**0.564219**	DIS	0.021	0.024	0.955	**−0.060111**
V	0.923	0.047	0.03	**0.658112**	NKE	0.386	0.04	0.575	**0.23267**
AXP	0.952	0.031	0.016	**0.676357**	WMT	0.94	0.036	0.023	**0.668724**
TRV	0.218	0.7	0.081	**0.294586**	KO	0.744	0.045	0.211	**0.516741**
AAPL	0.788	0.056	0.156	**0.554252**	CSCO	0.012	0.018	0.97	**−0.069042**
IBM	0.949	0.039	0.012	**0.676266**	DOW	0.951	0.035	0.014	**0.677014**
JPM	0.187	0.792	0.021	**0.297183**	VZ	0.326	0.039	0.636	**0.185025**
HON	0.941	0.028	0.032	**0.666625**	INTC	0.556	0.021	0.423	**0.482997**

We computed $\gamma_{i}$ using the Analytic Hierarchical Process (AHP) approach and outputs FinBERT model. The AHP approach is a multi-criteria decision-making strategy that assists in choosing the optimal choice from severalAlso, using the AHP method explained options based on various factors. This approach works well in unique circumstances where making decisions necessitates weighing a variety of intricate attributes. Saaty [130] was the first to suggest this approach in 1980. Saaty’s AHP is a widely used technique for prioritizing the basic challenges in a complicated situation [131]. We determined the weights of the three criteria: positive, negative, and neutral, utilizing this procedure, and subsequently calculated the $\gamma_{i}$ value by multiplying this vector with the output of the FeinBERT model. We have evaluated the value of preferences according to expert opinion, with positive to negative equal to 7, positive to neutral equal to 4, and neutral to negative equal to 3. The findings of this method are as follows:

Table 6 presents the decision matrix constructed as input for the AHP. This matrix captures the pairwise comparisons of the decision criteria, allowing for the quantification of subjective judgments in a structured format. It serves as the foundational step in deriving the normalized weights of the objective functions, which reflect the relative importance assigned to each criterion by the decision-maker. These weights are critical in guiding the optimization process and ensuring alignment with specific investment goals or risk preferences. Following this, Table 7 reports the outcomes of the AHP-based analysis. It presents the calculated weights, the corresponding scores for each portfolio alternative, and the resulting optimal portfolio configurations. The results are disaggregated by investor profile, enabling a nuanced understanding of how varying preferences such as risk tolerance or return expectations impact portfolio composition. This comprehensive presentation of results highlights the effectiveness of the proposed model in integrating multi-criteria decision-making with portfolio optimization.

**Table 6:** Decision matrix.

	Positive	Negative	Neutral
**Positive**	1	7	4
**Negative**	0.14	1	0.33
**Neutral**	0.25	3	1

**Table 7:** Demonstrating the resultant weights.

Cat.	Priority	Rank	(+)	(-)	CR
**Positive**	70.50%	1	12.60%	12.60%	3.4%
**Negative**	8.40%	3	1.50%	1.50%	
**Neutral**	21.10%	2	3.80%	3.80%	

It is important to highlight that the derived weight vector for the positive, neutral, and negative criteria is (0.705, 0.211, 0.084), respectively. Additionally, the Consistency Ratio (CR) obtained for the pairwise comparison matrix is 3.4%. In the context of the Analytic Hierarchy Process (AHP), the CR serves as an essential measure to evaluate the logical consistency of the judgments made during the pairwise comparison process. Given the subjective nature of human assessments, some degree of inconsistency is expected. However, a CR value below the widely accepted threshold of 10% indicates that the comparisons are sufficiently consistent and reliable for decision-making purposes. Therefore, with a CR of 3.4%, we can be confident in the robustness and credibility of the resulting weight vector. Letting *SAᵢ* denote the sentiment vector for the ith asset and *Q* the weight vector, the outlook score $\gamma_{i}$ is calculated using the element-wise product: $\gamma_{i}$ *= SAᵢ* ⊙ *Q*. An illustrative example follows:


$$\begin{array}{c} \gamma_{UNH}=[\text{0}.\text{7}\text{0}\text{5}\;\text{0}.\text{0}\text{8}\text{4}\;\text{0}.\text{2}\text{1}\text{1}]⊙[\text{0}.\text{3}\text{0}\text{7}\;\text{0}.\text{1}\text{0}\text{1}\;\text{0}.\text{5}\text{9}\text{2}]=\text{0}.\text{1}\text{8}\text{8}\text{0}\text{1}\text{8} \end{array}$$

### 5.3. Details on usage LSTM model

In this study, a LSTM model is employed to forecast two critical financial indicators: the expected return and the associated risk for each asset. These predicted values are represented within the proposed model as $\mu_{i}$ (expected return) and $\delta_{ij}$ (risk or variance-covariance elements between assets). The LSTM model is particularly well-suited for this task due to its ability to capture temporal dependencies and nonlinear patterns in financial time-series data. The specific architecture and configuration details of the implemented LSTM model, including the number of layers, units, dropout rates, and other training parameters, are summarized in Table 8.

**Table 8:** LSTM model summary.

Layer(type)	Output Shape	Param #
lstm (LSTM)	(None, 60, 128)	66,660
dropout(Dropout)	(None, 60, 128)	0
lstm_1(LSTM)	(None, 60, 64)	49,408
dropout_1 (Dropout)	(None, 60, 64)	0
lstm_2 (LSTM)	(None, 32)	12,416
dropout_2 (Dropout)	(None, 32)	0
dense(Dense)	(None, 1)	33
Total params: 385,253 (1.47 MB) Trainable params: 128,417 (501.63 KB) Non-trainable params: 0 (0.00 B) Optimizer params: 256,836 (1003.27 KB)		

**Fig 6:**
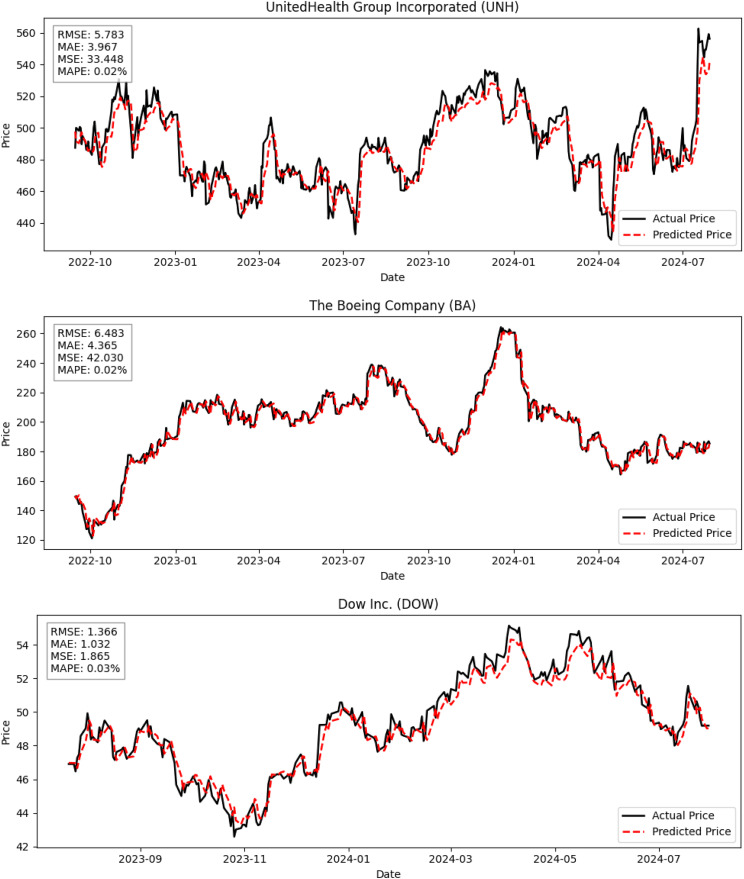
Showing examples of the LSTM model performance and comparing actual price with predicted price for three companies UNH, BA, DOW.

The developed model consists of an input layer, an output layer, and two hidden layers. Furthermore, two dropout layers have been added to mitigate the potential for overfitting. Here, a dropout rate of 20% is assumed that it is applied to the hidden layers’ input and functions by randomly deactivating 20% of the input units. In addition, a rate of 20% was used to divide the data into two categories: train and test data. The proposed model has shown acceptable performance in price prediction. Fig 6 displays the results of the proposed model’s performance as an example of all DJIA companies. The hyperparameters of the LSTM model, including the number of layers, the number of units per layer, dropout rates, batch size, and learning rate, were determined through a manual trial-and-error approach. Various configurations were tested based on values commonly used in related literature and prior experience with time-series forecasting tasks. The performance of each configuration was evaluated using validation data and standard metrics such as RMSE and MAE. The final set of hyperparameters was selected based on the best overall performance, balancing accuracy, model complexity, and overfitting prevention.

### 5.4. Implementation modified model

Given that most companies’ financial reports are published in late April or early May, in this study, the three-month period of May, June, and July has been considered for investment. Therefore, using the LSTM model, the price has been predicted for these three months, and finally the expected return and covariance matrix have been obtained. The expected return is calculated using the following formula.


$$\begin{array}{c} \overset{i}{\underset{t}{R}}=\frac{\overset{i}{\underset{t}{p}}-\overset{i}{\underset{t-\text{1}}{p}}}{\overset{i}{\underset{t-\text{1}}{p}}} \end{array}$$ (28)

Where $\overset{i}{\underset{t}{R}}$ represents the return on the asset *i* in period *t*. Also, $\overset{i}{\underset{t}{p}}$ represents the price of asset *i* in period *t* and $\overset{i}{\underset{t-\text{1}}{p}}$ represents the price of asset *i* in period *t-1*. The average expected return obtained for DJIA companies for *t*he first quarter of 2024 is shown in Table 9. The covariance matrix is also given in Table 10.

**Table 10:** Covariance matrix.

	UNH	GS	MSFT	HD	CAT	AMGN	MCD	CRM	V	AXP	TRV	AAPL	IBM	JPM	HON	AMZN	PG	JNJ	BA	CVX	MMM	MRK	DIS	NKE	WMT	KO	CSCO	DOW	VZ	INTC
UNH	1.013	0.169	−0.143	0.271	0.333	0.252	0.338	0.363	0.127	0.210	0.153	0.014	0.235	0.126	0.063	−0.222	0.095	0.211	0.264	0.233	0.357	0.069	−0.039	−0.194	0.121	0.254	0.282	0.203	0.304	0.037
GS	0.169	0.494	0.037	0.119	0.356	0.209	−0.043	0.261	0.219	0.350	0.122	0.093	0.229	0.372	0.080	0.059	0.008	0.029	0.360	0.164	0.157	0.043	0.059	−0.197	0.029	0.017	0.054	0.201	0.045	0.150
MSFT	−0.143	0.037	0.342	−0.005	0.038	0.035	−0.140	0.280	0.112	0.046	−0.033	0.260	−0.001	−0.031	0.128	0.236	0.010	−0.004	−0.096	−0.087	−0.360	0.016	−0.003	−0.021	0.102	−0.127	−0.089	0.041	−0.154	0.312
HD	0.271	0.119	−0.005	0.652	0.313	0.211	0.299	−0.086	0.187	0.179	0.256	0.232	0.145	0.078	0.205	−0.116	0.033	0.012	0.014	0.212	0.339	−0.064	0.185	0.106	0.034	0.023	0.197	0.299	0.132	0.233
CAT	0.333	0.356	0.038	0.313	0.936	0.210	0.098	0.477	0.314	0.428	0.307	0.089	0.382	0.271	0.087	0.108	0.029	0.128	0.354	0.438	0.458	−0.107	0.233	−0.036	−0.031	0.077	0.235	0.468	0.215	0.158
AMGN	0.252	0.209	0.035	0.211	0.210	0.677	0.208	0.338	0.226	0.161	0.197	0.115	0.169	0.034	0.201	0.079	0.102	0.080	0.242	0.132	0.056	0.269	0.148	0.307	0.083	0.159	0.208	0.095	0.145	0.150
MCD	0.338	−0.043	−0.140	0.299	0.098	0.208	0.755	0.063	0.116	0.060	0.205	−0.037	0.009	0.021	0.281	−0.179	0.067	0.029	0.359	0.184	0.461	0.205	0.213	0.308	0.156	0.260	0.328	0.092	0.297	−0.022
CRM	0.363	0.261	0.280	−0.086	0.477	0.338	0.063	3.737	0.087	0.153	−0.083	−0.073	0.456	0.057	−0.082	0.662	0.138	0.292	0.888	−0.151	0.650	0.179	−0.150	−0.252	0.047	0.031	0.329	−0.040	−0.526	0.148
V	0.127	0.219	0.112	0.187	0.314	0.226	0.116	0.087	0.812	0.302	0.284	0.117	0.189	0.297	0.286	0.134	0.035	0.019	0.069	0.194	0.718	0.272	0.121	0.039	0.077	0.072	0.127	0.185	0.050	0.147
AXP	0.210	0.350	0.046	0.179	0.428	0.161	0.060	0.153	0.302	0.722	0.376	0.089	0.187	0.531	0.271	0.068	−0.019	0.000	0.472	0.259	0.267	−0.050	0.158	−0.193	0.112	0.040	0.137	0.285	0.132	0.267
TRV	0.153	0.122	−0.033	0.256	0.307	0.197	0.205	−0.083	0.284	0.376	0.658	0.039	0.211	0.331	0.212	−0.006	0.033	0.020	0.332	0.302	0.556	0.017	0.151	0.132	0.135	0.122	0.222	0.285	0.185	0.246
AAPL	0.014	0.093	0.260	0.232	0.089	0.115	−0.037	−0.073	0.117	0.089	0.039	0.534	0.016	0.014	0.164	0.128	−0.006	−0.068	0.006	−0.104	−0.273	−0.071	0.015	−0.148	0.107	−0.115	−0.043	0.124	−0.023	0.331
IBM	0.235	0.229	−0.001	0.145	0.382	0.169	0.009	0.456	0.189	0.187	0.211	0.016	0.678	0.195	−0.109	−0.038	0.032	0.092	0.251	0.223	0.140	0.099	0.037	−0.174	0.041	0.092	0.197	0.156	0.157	0.135
JPM	0.126	0.372	−0.031	0.078	0.271	0.034	0.021	0.057	0.297	0.531	0.331	0.014	0.195	0.918	0.178	0.021	−0.041	−0.027	0.396	0.270	0.387	−0.011	0.083	−0.325	0.040	0.008	0.249	0.186	0.104	0.148
HON	0.063	0.080	0.128	0.205	0.087	0.201	0.281	−0.082	0.286	0.271	0.212	0.164	−0.109	0.178	0.638	0.116	0.061	−0.024	0.321	0.026	−0.014	0.115	0.140	0.242	0.170	0.042	0.102	0.184	0.012	0.261
AMZN	−0.222	0.059	0.236	−0.116	0.108	0.079	−0.179	0.662	0.134	0.068	−0.006	0.128	−0.038	0.021	0.116	0.717	0.011	−0.019	0.613	−0.103	−0.021	0.034	0.148	−0.056	0.017	−0.089	−0.018	−0.044	−0.199	0.167
PG	0.095	0.008	0.010	0.033	0.029	0.102	0.067	0.138	0.035	−0.019	0.033	−0.006	0.032	−0.041	0.061	0.011	0.069	0.059	0.047	0.004	0.071	0.086	0.008	0.186	0.034	0.039	0.013	0.015	−0.002	0.041
JNJ	0.211	0.029	−0.004	0.012	0.128	0.080	0.029	0.292	0.019	0.000	0.020	−0.068	0.092	−0.027	−0.024	−0.019	0.059	0.146	−0.007	0.027	0.152	0.070	−0.064	0.068	0.014	0.047	0.016	0.055	−0.081	−0.009
BA	0.264	0.360	−0.096	0.014	0.354	0.242	0.359	0.888	0.069	0.472	0.332	0.006	0.251	0.396	0.321	0.613	0.047	−0.007	2.665	0.050	0.690	0.063	0.354	0.044	0.357	0.261	0.206	0.216	0.158	0.167
CVX	0.233	0.164	−0.087	0.212	0.438	0.132	0.184	−0.151	0.194	0.259	0.302	−0.104	0.223	0.270	0.026	−0.103	0.004	0.027	0.050	0.678	0.152	0.087	0.240	0.011	0.043	0.148	0.295	0.298	0.446	0.132
MMM	0.357	0.157	−0.360	0.339	0.458	0.056	0.461	0.650	0.718	0.267	0.556	−0.273	0.140	0.387	−0.014	−0.021	0.071	0.152	0.690	0.152	5.400	0.100	0.381	0.116	−0.116	0.320	0.311	0.067	0.058	−0.334
MRK	0.069	0.043	0.016	−0.064	−0.107	0.269	0.205	0.179	0.272	−0.050	0.017	−0.071	0.099	−0.011	0.115	0.034	0.086	0.070	0.063	0.087	0.100	0.793	−0.101	0.291	0.115	0.110	0.145	−0.159	0.045	−0.031
DIS	−0.039	0.059	−0.003	0.185	0.233	0.148	0.213	−0.150	0.121	0.158	0.151	0.015	0.037	0.083	0.140	0.148	0.008	−0.064	0.354	0.240	0.381	−0.101	1.317	0.195	0.105	0.078	0.071	−0.003	0.177	0.281
NKE	−0.194	−0.197	−0.021	0.106	−0.036	0.307	0.308	−0.252	0.039	−0.193	0.132	−0.148	−0.174	−0.325	0.242	−0.056	0.186	0.068	0.044	0.011	0.116	0.291	0.195	1.945	0.116	0.185	0.048	0.047	−0.038	−0.190
WMT	0.121	0.029	0.102	0.034	−0.031	0.083	0.156	0.047	0.077	0.112	0.135	0.107	0.041	0.040	0.170	0.017	0.034	0.014	0.357	0.043	−0.116	0.115	0.105	0.116	0.622	0.065	−0.109	0.005	0.070	0.333
KO	0.254	0.017	−0.127	0.023	0.077	0.159	0.260	0.031	0.072	0.040	0.122	−0.115	0.092	0.008	0.042	−0.089	0.039	0.047	0.261	0.148	0.320	0.110	0.078	0.185	0.065	0.273	0.196	0.049	0.281	−0.128
CSCO	0.282	0.054	−0.089	0.197	0.235	0.208	0.328	0.329	0.127	0.137	0.222	−0.043	0.197	0.249	0.102	−0.018	0.013	0.016	0.206	0.295	0.311	0.145	0.071	0.048	−0.109	0.196	0.691	0.059	0.352	−0.021
DOW	0.203	0.201	0.041	0.299	0.468	0.095	0.092	−0.040	0.185	0.285	0.285	0.124	0.156	0.186	0.184	−0.044	0.015	0.055	0.216	0.298	0.067	−0.159	−0.003	0.047	0.005	0.049	0.059	0.624	0.091	0.083
VZ	0.304	0.045	−0.154	0.132	0.215	0.145	0.297	−0.526	0.050	0.132	0.185	−0.023	0.157	0.104	0.012	−0.199	−0.002	−0.081	0.158	0.446	0.058	0.045	0.177	−0.038	0.070	0.281	0.352	0.091	0.974	0.037
INTC	0.037	0.150	0.312	0.233	0.158	0.150	−0.022	0.148	0.147	0.267	0.246	0.331	0.135	0.148	0.261	0.167	0.041	−0.009	0.167	0.132	−0.334	−0.031	0.281	−0.190	0.333	−0.128	−0.021	0.083	0.037	1.857

**Table 9:** Average expected return.

Symbol	Expected return	Symbol	Expected return	Symbol	Expected return
UNH	13.85941301	TRV	−0.919235677	MMM	28.52682974
GS	12.92496931	AAPL	24.83713778	MRK	−3.759016112
MSFT	4.846565159	IBM	13.0001469	DIS	−21.41006165
HD	6.660085142	JPM	9.165720866	NKE	−26.51578354
CAT	2.062356412	HON	5.241876028	WMT	13.3576116
AMGN	18.83334605	AMZN	2.270461984	KO	7.539513667
MCD	−7.640643322	PG	3.570682037	CSCO	−0.258030221
CRM	−3.660161408	JNJ	4.582189429	DOW	−6.719393763
V	−4.807473489	BA	11.26747383	VZ	1.489743388
AXP	3.780771889	CVX	−4.700601787	INTC	−6.531948329

Using equation 20, the ${k\;}_{i}$ values for the three objective functions of risk, return, and trend are obtained as $\left[\begin{array}{ccc} \text{1}\text{0}.\text{3}\text{6}\text{1}\text{3}\text{8} & \text{6}\text{5}.\text{5}\text{4}\text{4}\text{2}\text{4} & \text{2}.\text{7}\text{5}\text{8}\text{7}\text{4} \end{array}\right]$, respectively. Furthermore, to calculate ${\text{b}}_{i}$, the proposed model solution with a single objective function is used. This approach involves simplifying the multi-objective problem by focusing on one objective at a time, which allows for a simpler optimization process. The value of 𝐵 is then used as a reference point or benchmark to evaluate and compare the performance of the multi-objective solution in subsequent analysis. The outcomes are displayed in Table 11.

**Table 11:** Ideal values for each objective.

Objective	W	$b_{i}$
Return	$w_{\text{2}\text{1}}=\text{1}$	$b_{\text{1}}=Z_{\text{1}}=\text{2}\text{8}.\text{5}\text{2}\text{7}$
Outlook Trend	$w_{\text{3}}=\text{1}$	$b_{\text{2}}=z_{\text{2}}=\text{0}.\text{6}\text{8}\text{0}\text{1}\text{2}\text{2}$
Risk	$w_{\text{3}}=\text{0}.\text{0}\text{9}\text{9}$, $w_{\text{1}\text{2}}=\text{0}.\text{0}\text{6}\text{6}$, $w_{\text{1}\text{4}}=\text{0}.\text{0}\text{6}\text{3}$, $w_{\text{1}\text{6}}=\text{0}.\text{0}\text{2}\text{8}$, $w_{\text{1}\text{7}}=\text{0}.\text{4}\text{3}\text{7}$, $w_{\text{1}\text{8}}=\text{0}.\text{1}\text{5}\text{4}$, $w_{\text{2}\text{3}}=\text{0}.\text{0}\text{1}\text{7}$, $w_{\text{2}\text{6}}=\text{0}.\text{0}\text{9}\text{5}$, $w_{\text{2}\text{9}}=\text{0}.\text{0}\text{4}\text{1}$	$b_{\text{3}}=z_{\text{3}}=\text{0}.\text{0}\text{4}\text{1}$

Also, using the AHP method explained in Section 5.1, the weighted values of the objective functions for the different investor states of risk-averse, risk-taking, and trend-based with the expert opinion are shown in Table 12. The AHP process begins with defining the objective, identifying the criteria and sub criteria, and constructing a hierarchy. Next, pairwise comparisons are made between the criteria using a relative importance scale, which typically ranges from 1 (equal importance) to 9 (extreme importance). These comparisons are used to create a pairwise comparison matrix from which the weights of the criteria are calculated, often by normalizing the matrix and calculating the average of each row. Finally, a consistency ratio is calculated to ensure that the judgments are consistent. If this ratio is acceptable (usually less than 0.1), the weights obtained can be used in the decision-making process. Here, we weighted the three objectives according to expert opinion and formed optimal portfolios based on these results.

**Table 12:** Objective function weights in the goal programming technique for different types of investors.

No.	Decision matrix	Priority	Consistency Ratio	Variables	Auxiliary variable	GP Objective
**1**	$\left[\begin{array}{ccc} \text{1} & \text{1} & \text{1}\\ \text{1} & \text{1} & \text{1}\\ \text{1} & \text{1} & \text{1} \end{array}\right]$	$\left[\begin{array}{ccc} \text{3}\text{3}.\text{3}\text{3}\% & \text{3}\text{3}.\text{3}\text{3}\% & \text{3}\text{3}.\text{3}\text{3}\% \end{array}\right]$	0%	$w_{\text{1}\text{2}}=\text{0}.\text{8}\text{7}$, $w_{\text{2}\text{1}}=\text{0}.\text{0}\text{7}\text{2}$, $w_{\text{6}}=\text{0}.\text{0}\text{5}\text{8}$	$n_{re}=\text{3}.\text{7}\text{6}\text{9}$, $n_{dt}=\text{0}.\text{1}\text{4}\text{3}$, $p_{ri}=\text{0}.\text{3}\text{7}\text{1}$	0.048
**2**	$\left[\begin{array}{ccc} \text{1} & \text{1} & \text{1}\\ \text{1} & \text{1} & \text{2}\\ \text{1} & \text{0}.\text{5} & \text{1} \end{array}\right]$	$\left[\begin{array}{ccc} \text{3}\text{2}.\text{7}\% & \text{4}\text{1}.\text{3}\% & \text{2}\text{6}\% \end{array}\right]$	5.6%	$w_{\text{2}\text{1}}=\text{0}.\text{1}\text{0}\text{9}$, $w_{\text{1}\text{2}}=\text{0}.\text{8}\text{9}\text{1}$	$n_{re}=\text{3}.\text{2}\text{7}$, $n_{dt}=\text{0}.\text{1}\text{6}$, $p_{ri}=\text{0}.\text{3}\text{9}\text{4}$	0.048
**3**	$\left[\begin{array}{ccc} \text{1} & \text{4} & \text{3}\\ \text{0}.\text{2}\text{5} & \text{1} & \text{1}\\ \text{0}.\text{3}\text{3} & \text{1} & \text{1} \end{array}\right]$	$\left[\begin{array}{ccc} \text{6}\text{3}.\text{4}\% & \text{1}\text{7}.\text{4}\% & \text{1}\text{9}.\text{2}\% \end{array}\right]$	1%	$w_{\text{1}\text{2}}=\text{0}.\text{5}\text{4}\text{8}$, $w_{\text{1}\text{3}}=\text{0}.\text{0}\text{9}\text{7}$, $w_{\text{6}}=\text{0}.\text{2}\text{0}\text{6}$, $w_{\text{2}\text{1}}=\text{0}.\text{0}\text{6}\text{4}$, $w_{\text{2}\text{5}}=\text{0}.\text{0}\text{8}\text{5}$	$n_{re}=\text{6}.\text{8}\text{1}\text{9}$, $n_{dt}=\text{0}.\text{1}\text{0}\text{6}$, $p_{ri}=\text{0}.\text{2}\text{1}\text{2}$	0.038
**4**	$\left[\begin{array}{ccc} \text{1} & \text{1} & \text{0}.\text{1}\text{7}\\ \text{1} & \text{1} & \text{0}.\text{1}\text{2}\\ \text{6} & \text{8} & \text{1} \end{array}\right]$	$\left[\begin{array}{ccc} \text{1}\text{1}.\text{7}\% & \text{1}\text{0}.\text{7}\% & \text{7}\text{7}.\text{6}\% \end{array}\right]$	1%	$w_{\text{1}\text{3}}=\text{0}.\text{3}\text{1}\text{9}$, $w_{\text{2}\text{5}}=\text{0}.\text{2}\text{8}\text{6}$, $w_{\text{6}}=\text{0}.\text{3}\text{9}\text{5}$	$n_{re}=\text{1}\text{3}.\text{1}\text{2}\text{0}$, $n_{dt}=\text{0}.\text{0}\text{1}\text{9}$, $p_{ri}=\text{0}.\text{2}\text{5}\text{4}$	0.031
**5**	$\left[\begin{array}{ccc} \text{1} & \text{0}.\text{5} & \text{0}.\text{5}\\ \text{2} & \text{1} & \text{1}\\ \text{2} & \text{1} & \text{1} \end{array}\right]$	$\left[\begin{array}{ccc} \text{2}\text{0}\% & \text{4}\text{0}\% & \text{4}\text{0}\% \end{array}\right]$	0%	$w_{\text{2}\text{1}}=\text{0}.\text{0}\text{3}\text{3}$, $w_{\text{1}\text{2}}=\text{0}.\text{9}\text{6}\text{7}$	$n_{re}=\text{3}.\text{5}\text{6}\text{7}$, $n_{dt}=\text{0}.\text{1}\text{3}\text{6}$, $p_{ri}=\text{0}.\text{4}\text{4}\text{7}$	0.05
**6**	$\left[\begin{array}{ccc} \text{1} & \text{1} & \text{0}.\text{5}\\ \text{1} & \text{1} & \text{0}.\text{2}\\ \text{2} & \text{5} & \text{1} \end{array}\right]$	$\left[\begin{array}{ccc} \text{2}\text{2}.\text{5}\% & \text{1}\text{6}.\text{3}\% & \text{6}\text{1}.\text{2}\% \end{array}\right]$	9.8%	$w_{\text{1}\text{2}}=\text{0}.\text{3}\text{2}\text{0}$, $w_{\text{1}\text{3}}=\text{0}.\text{1}\text{4}\text{6}$, $w_{\text{6}}=\text{0}.\text{3}\text{9}\text{6}$ $w_{\text{2}\text{5}}=\text{0}.\text{1}\text{3}\text{8}$	$n_{re}=\text{9}.\text{3}\text{8}\text{4}$, $n_{dt}=\text{0}.\text{0}\text{5}\text{7}$, $p_{ri}=\text{0}.\text{2}\text{1}\text{7}$	0.041
**7**	$\left[\begin{array}{ccc} \text{1} & \text{2} & \text{2}\\ \text{0}.\text{5} & \text{1} & \text{1}\\ \text{0}.\text{5} & \text{1} & \text{1} \end{array}\right]$	$\left[\begin{array}{ccc} \text{5}\text{0}\% & \text{2}\text{5}\% & \text{2}\text{5}\% \end{array}\right]$	0%	$\;w_{\text{1}\text{2}}=\text{0}.\text{7}\text{1}\text{2}$, $\;w_{\text{2}\text{1}}=\text{0}.\text{0}\text{7}\text{7}$, $w_{\text{6}}=\text{0}.\text{2}\text{1}\text{1}$	$n_{re}=\text{4}.\text{6}\text{7}\text{0}$, $n_{dt}=\text{0}.\text{1}\text{3}\text{1}$, $p_{ri}=\text{0}.\text{2}\text{9}\text{8}$	0.044
**8**	$\left[\begin{array}{ccc} \text{1} & \text{0}.\text{2}\text{5} & \text{0}.\text{5}\\ \text{4} & \text{1} & \text{1}\\ \text{2} & \text{5} & \text{1} \end{array}\right]$	$\left[\begin{array}{ccc} \text{1}\text{4}.\text{9}\% & \text{4}\text{7}.\text{4}\% & \text{3}\text{7}.\text{6}\% \end{array}\right]$	5.6%	$w_{\text{2}\text{1}}=\text{0}.\text{0}\text{3}\text{9}$, $w_{\text{1}\text{2}}=\text{0}.\text{9}\text{6}\text{1}$	$n_{re}=\text{3}.\text{5}\text{4}\text{7}$, $n_{dt}=\text{0}.\text{1}\text{3}\text{8}$, $p_{ri}=\text{0}.\text{4}\text{4}\text{0}$	0.051
**9**	$\left[\begin{array}{ccc} \text{1} & \text{0}.\text{2} & \text{1}\\ \text{5} & \text{1} & \text{5}\\ \text{1} & \text{0}.\text{2} & \text{1} \end{array}\right]$	$\left[\begin{array}{ccc} \text{1}\text{4}.\text{3}\% & \text{7}\text{1}.\text{4}\% & \text{1}\text{4}.\text{3}\% \end{array}\right]$	0%	$w_{\text{2}\text{1}}=\text{0}.\text{2}\text{5}\text{9}$, $w_{\text{1}\text{2}}=\text{0}.\text{7}\text{4}\text{1}$	$n_{re}=\text{2}.\text{7}\text{3}\text{6}$, $n_{dt}=\text{0}.\text{2}\text{0}\text{7}$, $p_{ri}=\text{0}.\text{5}\text{0}\text{9}$	0.048
**10**	$\left[\begin{array}{ccc} \text{1} & \text{0}.\text{2} & \text{0}.\text{3}\text{3}\\ \text{5} & \text{1} & \text{3}\\ \text{3} & \text{0}.\text{3}\text{3} & \text{1} \end{array}\right]$	$\left[\begin{array}{ccc} \text{1}\text{0}.\text{5}\% & \text{6}\text{3}.\text{7}\% & \text{2}\text{5}.\text{8}\% \end{array}\right]$	4%	$w_{\text{2}\text{1}}=\text{0}.\text{1}\text{7}\text{5}$, $w_{\text{1}\text{2}}=\text{0}.\text{8}\text{2}\text{5}$	$n_{re}=\text{3}.\text{0}\text{9}\text{5}$, $n_{dt}=\text{0}.\text{1}\text{8}\text{1}$, $p_{ri}=\text{0}.\text{4}\text{0}\text{9}$	0.051

As shown in Table 11, the CR value for all three cases was less than 10%, so the calculated weights are acceptable. To calculate the $b_{i}$ values, we solved the initial model with each of the objective functions alone, the results of which are shown in Table 12.

## 6. Discussion

This section consists of two parts: first, a review and discussion of the results obtained in the previous section; second, a comparison and review of the proposed approach with previous studies. Therefore, we will first carefully analyze and interpret the findings presented in Table 12. To ensure a comprehensive assessment, we will evaluate the performance of the proposed model using five essential financial indicators: beta, Sharpe ratio, Treynor ratio, and Sortino ratio. These metrics provide distinct insights into different dimensions of risk-adjusted performance and allow for a deeper and more detailed examination of the results. Then, the proposed model will also be compared with previously presented models on the stock portfolio problem.

### 6.1. *Review and discussion of the results*

**Beta:** William Sharpe [132] proposed that systematic risk can be quantified using the beta coefficient (β). Beta acts as a measure of a security’s responsiveness to changes in the broader market’s performance. It quantifies the extent to which an asset’s returns correlate with those of the market portfolio. Specifically, the beta coefficient assesses how an asset’s returns react to variations in the market’s returns, calculated by analyzing the relationship between the asset’s return sensitivity and the market’s return sensitivity. Mathematically, beta is defined as the ratio of the covariance of the security’s returns and the market portfolio’s returns to the variance of the market portfolio’s returns, represented as follows:


$$\begin{array}{c} \beta_{i}=\frac{Cov\left(R_{asset},R_{market}\right)}{\overset{\text{2}}{\underset{market}{\sigma }}} \end{array}$$ (29)

The portfolio beta is calculated from the following formula:


$$\begin{array}{c} \;\beta =\sum_{i=\text{1}}^{n}\beta_{i}w_{i} \end{array}$$ (30)

Where *n* represents the number of stocks in the portfolio and $w_{i}$ represents the weight of the *ith* asset.

**Sharp ratio:** The Sharpe Ratio (SR), introduced by Sharpe in 1964 [132], serves as a standardized and comprehensive metric for evaluating fund performance. It effectively accounts for both returns and associated risks, mitigating the impact of risk factors on performance assessments. A higher SR value indicates a superior risk-adjusted return. The Sharpe Ratio is computed using the following formula:


$$\begin{array}{c} SR=\frac{E(R)-R_{f}}{\sigma (R)} \end{array}$$ (31)

Where E(R) represents the expected return, σ(R) denotes the standard deviation of the returns, and $R_{f}$ is the risk-free rate.

**Treynor ratio:** The Treynor Ratio is a prominent and important performance metric in financial literature. Commonly known as a “reward-to-volatility” metric, it evaluates the return produced by a portfolio in relation to the risk assumed, particularly emphasizing systematic risk, denoted as beta (β). This ratio is a one-dimensional metric that accounts for both residual return and the beta coefficient. Beta, denoted by the Greek letter β\beta, is a measure of the sensitivity of a portfolio’s returns to fluctuations in the overall market. It quantifies the degree to which the portfolio’s performance is affected by systematic market risk. Beta is typically calculated using a market index or a benchmark portfolio as a reference [133]. To calculate the Treynor Ratio, the residual return is divided by the portfolio’s beta, as shown in the following formula:


$$\begin{array}{c} TR=\frac{E(R)-R_{f}}{\beta } \end{array}$$ (32)

**Sortino ratio:** The Sortino ratio is a variation of the Sharpe ratio, but it replaces standard deviation with downside deviation as the measure of risk. Unlike the Sharpe ratio, which considers all fluctuations in return, the Sortino ratio focuses solely on returns that fall below a user-defined target or required rate of return, treating only those negative deviations as risky [134]. The formula for calculating the Sortino ratio is as follows:


$$\begin{array}{c} SR=\frac{E(R)-R_{f}}{\sigma_{d}} \end{array}$$ (33)

Where $\sigma_{d}$ represents the downward deviation of the portfolio. And it is calculated from the following formula:


$$\begin{array}{c} \sigma_{d}=\sqrt{\frac{\text{1}}{n}\sum_{i}^{n}{\left(\text{min}\left(R_{i}-R_{f},\text{0}\right)\right)}^{\text{2}}} \end{array}$$ (34)

For each of the proposed portfolios listed in Table 12, the following performance measures have been calculated to evaluate them. These calculations provide a detailed and comprehensive assessment of the risk-return profile for each portfolio and provide key insights into their potential performance under different market conditions. The performance measures include expected return, portfolio variance, Sharpe ratio, beta index, Treynor ratio, and Sortino ratio, all of which serve as critical indicators for assessing the profitability and risk associated with each portfolio. A summary of the results, for further analysis and comparison, is provided in Table 13.

**Table 13:** Performance measures results for portfolios.

No.	Return	Variance	Beta	Sharp ratio	Treynor ratio	Sortino ratio
1	24.755	0.8562	1.186	21.3498	16.6621	87.1528
2	25.239	0.4271	1.214	30.9678	20.7929	85.1203
3	21.713	0.2263	0.980	35.1307	22.1514	103.8307
4	15.407	0.2915	0.612	19.2736	25.1654	55.0311
5	24.959	0.4796	1.232	28.8192	20.2575	78.3673
6	19.147	0.2547	0.810	28.0311	23.6438	91.3168
7	23.854	0.3341	1.086	32.6210	21.9557	101.1982
8	24.981	0.4728	1.231	29.0587	20.2992	78.8191
9	25.793	0.5399	1.178	28.2962	21.8984	97.4635
10	25.483	0.4414	1.198	30.8304	21.2711	91.1750

According to the results presented in Table 13, various strategies can be customized to suit investors with diverse risk appetites. For instance, if return is the only concern, portfolio number 9 stands out as the top choice, offering the highest return but also carrying a significant level of risk. However, a deeper analysis of the results reveals the following key insights:

- The beta index is a critical metric for evaluating a portfolio’s systematic risk, as it reflects the portfolio’s sensitivity to overall market fluctuations. A lower beta value is generally more favorable for risk-averse investors, indicating reduced exposure to market volatility. Among the proposed portfolios, portfolios 4 and 6 demonstrated the lowest beta ratios, making them particularly appealing from a risk-management perspective. However, there is a notable trade-off between risk and return. While portfolio 4 achieved the lowest beta index, it also yielded the lowest return, potentially limiting its appeal for investors focused on maximizing profitability. In contrast, portfolio 6 offered a more favorable balance, combining a low beta ratio with a comparatively higher return, thereby emerging as a stronger option for those seeking moderate risk alongside improved returns. Furthermore, during the optimization process, these portfolios were assigned a higher weight under objective number three, which prioritizes the desirability of future price trends. This allocation highlights the model’s emphasis on trend-following performance and reinforces the suitability of Portfolio 6 for trend-based investment strategies. These findings highlight the importance of carefully balancing risk and return according to investor preferences, as well as the significant influence of trend desirability in determining optimal portfolio configurations.
- The Sharpe ratio, a well-known indicator of risk-adjusted return, offers important insights into the performance of the proposed portfolios. Based on the calculated Sharpe ratios, Portfolio 3 exhibits the highest value, indicating that it delivers a superior return relative to its level of risk. This makes Portfolio 3 an attractive option for investors seeking to maximize returns while maintaining a favorable risk-reward balance. Conversely, Portfolio 4 displays the lowest Sharpe ratio, suggesting that its return is comparatively lower relative to the risk undertaken. While this portfolio may appeal to highly risk-averse investors prioritizing minimal exposure to volatility, its limited return potential may not suit those seeking significant gains. These findings underline the importance of tailoring portfolio selection to align with an investor’s specific risk tolerance and investment objectives. For risk-tolerant investors, portfolios with higher Sharpe ratios, such as Portfolios 2, 10 and 3, may be more appealing as they emphasize higher returns for a given level of risk. On the other hand, risk-averse investors might prioritize portfolios with lower risk, even if the risk-adjusted returns are less favorable. This analysis highlights the trade-offs inherent in portfolio selection and underscores the versatility of the proposed model in accommodating diverse investor profiles. By providing a range of portfolio options with varying risk-return characteristics, the model supports informed decision-making tailored to individual preferences and market conditions.
- The Treynor ratio is an important performance metric that evaluates a portfolio’s excess return in relation to its systematic risk, or market risk. It is calculated using the portfolio’s beta, which measures the portfolio’s sensitivity to overall market movements. A higher Treynor ratio suggests that the portfolio is generating greater returns for each unit of systematic risk, making it a crucial measure of risk-adjusted performance. In the proposed portfolios, Portfolio 4 stands out with the highest Treynor ratio, signaling that it delivers the most return relative to its systematic risk. However, it is crucial to emphasize that Portfolio 4 also has the lowest overall return, which suggests that while it is optimized for managing market risk, it may not provide the level of returns sought by more aggressive investors. This makes Portfolio 4 particularly suitable for risk-averse investors, who are focused on minimizing exposure to market fluctuations while choosing reduced returns in return for enhanced stability. Portfolio 6, on the other hand, demonstrates a relatively high Treynor ratio as well, indicating a favorable return in relation to systematic risk. In addition, Portfolio 6 offers a higher return compared to Portfolio 4, this makes it a more attractive choice for investors looking for a balance between risk and reward. This portfolio may be considered ideal for normal investors who are comfortable with some level of market risk but still prioritize a reasonable level of return. Finally, Portfolio 9 is also recommended for risk-taking investors due to its relatively high level of systematic risk and its relatively high return.
- The Sortino ratio is an essential measure for assessing portfolio performance, particularly when the primary concern is managing downside risk—the potential for negative returns that could erode capital. This metric is particularly useful for investors aiming to limit losses while maximizing returns, especially in volatile market environments where shielding against substantial drawdowns is critical. Among the proposed portfolios, Portfolios 3 and 7 exhibit higher Sortino ratios compared to the other options, signaling better control over downside risk. However, despite their relatively favorable risk profiles, these portfolios do not offer the highest returns, making them less appealing for investors prioritizing return potential. In contrast, Portfolio 9 stands out as a more balanced option. It achieves a relatively high Sortino ratio, suggesting effective downside protection, while also delivering more favorable returns than Portfolios 3 and 7. This combination of strong risk management and higher returns makes Portfolio 9 a more attractive choice for investors who aim to optimize both performance and downside risk.

The choice of an optimal portfolio for investors is shaped by several factors, with the final decision largely based on the individual preferences and objectives of the investor. One of the most critical factors in portfolio selection is the amount of risk an investor is willing to take, which can vary significantly across individuals. Risk tolerance is a personal characteristic that shapes how much volatility or potential loss an investor is prepared to endure in pursuit of higher returns. As a general principle, investors who accept higher levels of risk are often rewarded with the possibility of greater returns, although this comes with the potential for larger fluctuations in the portfolio’s value. Another important factor considered in this study is the appropriate trend forecast of future price movements, which can provide valuable insight into how a portfolio will perform under prevailing conditions. Investors who can identify favorable trends may be better positioned to capitalize on market dynamics. To support investors in making better-informed decisions, this study analyzed the suitability of various portfolios in relation to future price movements. The findings of this analysis are presented in Table 12, where the performance of various portfolios is evaluated with respect to the consideration of price movement trend forecasts. By considering risk tolerance, expected returns, and trend desirability, investors can more effectively align their portfolio choices with investment objectives and market expectations. This three-pronged approach allows for a more comprehensive assessment and helps investors strike a balance between optimizing potential returns and controlling risk exposure. Ultimately, the decision-making process becomes more tailored, ensuring that investors select portfolios that align not only with their personal risk preferences but also with the broader company’s outlook.

### 6.2. *Comparison with other portfolio optimization model*

In this paper, we tried to present a new model in which we used new neural network models and artificial intelligence tools. In this model, we tried to add a new criterion as the outlook of the possible future price movement to improve the model results. Our proposed model included two types of text and price data, which we calculated the price data using the LSTM model, and for text data, we collected the quarterly reports of DJIA companies from the Yahoo Finance website and extracted the companies’ outlook for the next quarter using the Cider artificial intelligence tool. To convert this text data into a score, we used the FinBERT model and performed sentiment analysis on the outlooks, and considering that the output of the FinBERT model was a vector (positive, neutral, negative), we used the AHP method to convert this vector into a score and finally extracted the outlook score, this process can be seen in Fig 5. In fact, we tried to present a State-of-the-Art model. The topic of sentiment analysis in most previous models has been that first, sentiment analysis was performed on Twitter data, websites, and social networks, and second, the results were used as one of the features for price prediction. While in our study, we have used data from companies’ quarterly reports with an innovative approach. Among the previous studies, the following can be mentioned:

Colasanto et al. [135] In one study, sentiment scores, particularly polarity values, were incorporated into stock price forecasting by introducing them as an additional “view” in the Black-Letterman framework. These scores were extracted from textual analysis of Financial Times articles and were associated with various events, both favorable and unfavorable, that affected specific stocks. The key distinction of the present study is in the application of the FinBERT model, which is used not only as a predictive feature but also to extract insights into future performance from corporate disclosures. Unlike previous approaches that used sentiment as a direct input for price forecasting, our method uses FinBERT to assess the outlook of companies, thereby informing investment decisions with a more interpretative and qualitative layer. Leow et al. [136] introduced two novel models SAW and SMPT which incorporate real-time market sentiment derived from Twitter data, analyzed using BERT. These models adjust portfolio allocation weights based on the extracted sentiment signals. To optimize their performance, genetic algorithms are employed with objectives such as maximizing cumulative returns and minimizing volatility. In contrast, the present study relies on sentiment extracted from corporate quarterly reports to assess future outlooks, offering a more fundamental and document-based approach to sentiment analysis. Day and Li [137] examined the influence of various financial information sources on investment decisions and explored how deep learning techniques can enhance the accuracy of financial news classification. Their empirical findings demonstrated that the type of financial source significantly affects investor behavior and decision-making outcomes, and that classification performance benefits from the application of deep learning models. However, unlike the current study, their research did not address portfolio optimization; instead, it focused solely on analyzing the effects of information quality and classification on investment behavior. Fatouros et al. [138] conducted a pioneering study exploring the capabilities of large language models specifically ChatGPT-3.5 in the context of financial sentiment analysis, with a focus on the FOREX market. Employing a goal-free prompting strategy, they evaluated various ChatGPT prompts on a substantial dataset of FOREX related news headlines. Model performance was assessed using standard evaluation metrics, including precision, recall, F1-score, and MAE for sentiment classification. Notably, the study does not propose a structured or model-driven investment framework; rather, it centers on assessing the viability of LLMs for sentiment detection. Seshakagari et al. [139] employed three advanced models FinBERT, GPT-4, and T5 for the task of financial sentiment classification. By evaluating these models using precision, recall, and F1 score, the study contributes to the field by offering comparative insights that guide the selection of the most suitable model depending on specific application needs in financial sentiment analysis. However, similar to other works in this domain, their study does not extend to portfolio construction or optimization, focusing solely on the classification task.

Generally, the proposed model distinguishes itself through two notable and innovative features. First, it integrates qualitative news data extracted from companies’ quarterly reports, allowing the model to capture sentiment, forward-looking statements, and performance insights that are not typically reflected in historical price data alone. This incorporation of textual and contextual information enhances the depth of the analysis and supports more informed investment decisions. Second, the model introduces a three-objective optimization framework, expanding beyond the traditional focus on return maximization and risk minimization. The third objective specifically evaluates the future outlook of companies, derived from financial reports and qualitative assessments. This added dimension allows the model to account for anticipated growth, market positioning, and management guidance factors that are increasingly relevant in dynamic and uncertain market environments. The effectiveness of this approach is demonstrated in Table 12, which presents the performance metrics of the optimized portfolios. Notably, the Sharpe ratios of portfolios that assign greater weight to the third objective the forward-looking outlook show superior risk-adjusted returns. This indicates that incorporating qualitative future expectations alongside quantitative financial data can lead to more robust and strategically balanced investment outcomes.

## 7. Findings and conclusion

In this section, we have analyzed the key findings derived from the proposed model, highlighting its strengths and potential areas for further refinement. While the model has demonstrated promising results in portfolio management, LSTM model, and sentiment analysis, there remain opportunities to improve its performance and broaden its scope of application.

This study explored the potential of using the rich information contained in financial statements to guide long-term investment decisions. We introduced a comprehensive three-stage model for portfolio optimization that integrates sentiment analysis, price forecasting, and multi-objective optimization. The model is organized in the following manner: the first stage involves sentiment analysis of companies’ quarterly reports to gauge market sentiment and predict trend movements using FinBERT model and Sider AI, the second stage uses a LSTM model to predict prices, and the third stage uses a multi-objective model to optimize the portfolio. The results of this research show that sentiment analysis of quarterly reports can provide valuable insights and correctly predict future price movements approximately 70% of the time. It is essential to highlight that this prediction refers to the general direction of price changes – whether bullish, bearish or stagnant – rather than specific numerical price values. The LSTM model, in the second stage, was used to cover this issue by combining historical data and capturing time dependencies to estimate the magnitude of price movements. To validate the effectiveness of the proposed model, we used real-world data from companies listed in the DJIA. This practical validation showed that the model is not only theoretically sound but also capable of providing practical insights into real-world investment.

Although the suggested model has shown encouraging outcomes, there remain several opportunities to enhance its precision through additional improvements, robustness, and practical applicability in dynamic market conditions. The current approach effectively integrates sentiment analysis, price forecasting, and portfolio optimization, but future research can build on this foundation by addressing key areas for improvement. One important direction is incorporating technical analysis data, which could enhance predictive accuracy and provide deeper insights for decision-making. Additionally, the inclusion of a multi-period framework and dynamic portfolio rebalancing would allow the model to better adapt to evolving market conditions. It would also be valuable to integrate investor preferences and risk tolerances, providing a more personalized approach to portfolio optimization. Further, exploring hybrid and more sophisticated forecasting models could improve price prediction accuracy, while considering the implications of a two-sided market and incorporating short selling would offer more realistic and flexible strategies. The proposed model can also be implemented on data from larger open indices such as Nasdaq and S&P 500. Finally, expanding the model to account for constraints such as cardinality, transaction costs, stock dependencies, and liquidity issues would enhance its robustness and make it more aligned with real-world investment scenarios. Together, these improvements could transform the model into a more comprehensive, adaptable, and practical tool for long-term investment decision-making.

### Limitations

While the selection of DJIA companies provides a stable and well-documented dataset for model development and validation, it also introduces a limitation regarding the generalizability of the results. The DJIA includes only 30 large-cap, U.S.-based blue-chip companies, which may not fully represent the broader equity market, including small-cap stocks, emerging industries, or firms operating in different regulatory or economic environments. As such, the findings and model performance observed in this study may not directly translate to other market segments or international contexts. Future research should consider applying and testing the model on more diverse datasets, encompassing a wider range of firms across different indices, sectors, and regions

## References

[1] Kolm PN, Tütüncü R, Fabozzi FJ. 60 Years of portfolio optimization: Practical challenges and current trends. European Journal of Operational Research. 2014;234(2):356–71. doi: 10.1016/j.ejor.2013.10.060

[2] Markowitz H. Modern portfolio theory. Journal of Finance. 1952;7(11):77–91.

[3] Amini A, Rahnama G, Alinezhad A. Ranking and managing stock in the stock market using fundamental and technical analyses. 2015;4(3).

[4] Kumar RR, Ghanbari H, Stauvermann PJ. Application of a robust maximum diversified portfolio to a small economy’s stock market: an application to fiji’s south pacific stock exchange. JRFM. 2024;17(9):388. doi: 10.3390/jrfm17090388

[5] Leow EKW, Nguyen BP, Chua MCH. Robo-advisor using genetic algorithm and BERT sentiments from tweets for hybrid portfolio optimisation. Expert Systems with Applications. 2021;179:115060. doi: 10.1016/j.eswa.2021.115060

[6] Larni-Fooeik A, Ghanbari H, Shabani M, Mohammadi E. Bi-objective portfolio optimization with mean-CVaR model: An ideal and anti-ideal compromise programming approach. In: Yazdi M, editor. Progressive decision-making tools and applications in project and operation management. Cham: Springer Nature Switzerland. 2024. p. 69–79. doi: 10.1007/978-3-031-51719-8_5

[7] Fooeik AML, Ghanbari H, Sadjadi SJ, Mohammadi E. Behavioral finance biases: a comprehensive review on regret approach studies in portfolio optimization. International J Industrial Engineering. 2024;35(1):1–23.

[8] Colasanto F, Grilli L, Santoro D, Villani G. BERT’s sentiment score for portfolio optimization: a fine-tuned view in Black and Litterman model. Neural Comput Appl. 2022;34(20):17507–21. doi: 10.1007/s00521-022-07403-1 35669537 PMC9150638

[9] Medhat W, Hassan A, Korashy H. Sentiment analysis algorithms and applications: a survey. Ain Shams Engineering Journal. 2014;5(4):1093–113. doi: 10.1016/j.asej.2014.04.011

[10] Al-Qablan T, Mohd Noor MH, Al-Betar M, Khader AT. A survey on sentiment analysis and its applications. Neural Computing and Applications. 2023;35:1–35. doi: 10.1007/s00521-023-08941-y

[11] Chopra A, Prashar A, Sain C. Natural Language Processing. 2013.

[12] Vaswani A. Attention is All You Need. 2017.

[13] Devlin J, Chang M-W, Lee K, Toutanova K. BERT: Pre-training of Deep Bidirectional Transformers for Language Understanding. arXiv. 2018. doi: 10.48550/arXiv.1810.04805

[14] Radford A, Narasimhan K, Salimans T, Sutskever I. Improving Language Understanding by Generative Pre-Training. N/A. 2018;N/A(N/A):N/A.

[15] Rumelhart DE, Hinton GE, Williams RJ. Learning representations by back-propagating errors. Nature. 1986;323(6088):533–6. doi: 10.1038/323533a0

[16] Cho K, et al. Learning phrase representations using RNN encoder-decoder for statistical machine translation. 2014. doi: 10.48550/arXiv.1406.1078

[17] Araci D. FinBERT: Financial sentiment analysis with pre-trained language models. 2019. doi: 10.48550/arXiv.1908.10063

[18] Deboeck GJ. Trading on the Edge: Neural, Genetic, and Fuzzy Systems for Chaotic Financial Markets. John Wiley & Sons; 1994.

[19] Wang J-Z, Wang J-J, Zhang Z-G, Guo S-P. Forecasting stock indices with back propagation neural network. Expert Systems with Applications. 2011;38(11):14346–55.

[20] Ghanbari H, Mohammadi E, Barzinpour F, Paeizi A. The mean-variance cardinality constrained portfolio selection using an enhanced genetic algorithm with a novel crossover operator. Journal of Industrial and Systems Engineering. 2024;16(3):1–19.

[21] Ma Y, Han R, Wang W. Portfolio optimization with return prediction using deep learning and machine learning. Expert Systems with Applications. 2021;165:113973. doi: 10.1016/j.eswa.2020.113973

[22] Emir S. Predicting the Istanbul Stock Exchange Index Return Using Technical Indicators: A Comparative Study. International Journal of Finance & Banking Studies. 2013;2(3):Art. no. 3. doi: 10.20525/ijfbs.v2i3.158

[23] Matías JM, Reboredo JC. Forecasting performance of nonlinear models for intraday stock returns. Journal of Forecasting. 2012;31(2):172–88. doi: 10.1002/for.1218

[24] Rasel RI, Sultana N, Meesad P. An efficient modelling approach for forecasting financial time series data using support vector regression and windowing operators. IJCISTUDIES. 2015;4(2):134. doi: 10.1504/IJCISTUDIES.2015.071180

[25] Ballings M, Van den Poel D, Hespeels N, Gryp R. Evaluating multiple classifiers for stock price direction prediction. Expert systems with Applications. 2015;42(20):7046–56.

[26] Patel J, Shah S, Thakkar P, Kotecha K. Predicting stock and stock price index movement using Trend Deterministic Data Preparation and machine learning techniques. Expert Systems with Applications. 2015;42(1):259–68. doi: 10.1016/j.eswa.2014.07.040

[27] Chong E, Han C, Park FC. Deep learning networks for stock market analysis and prediction: methodology, data representations, and case studies. Expert Systems with Applications. 2017;83. doi: 10.1016/j.eswa.2017.04.030

[28] Hochreiter S, Schmidhuber J. Long short-term memory. Neural Computation. 1997;9(8):1735–80. doi: 10.1162/neco.1997.9.8.1735 9377276

[29] Fischer T, Krauss C. Deep learning with long short-term memory networks for financial market predictions. European Journal of Operational Research. 2018;270(2):654–69. doi: 10.1016/j.ejor.2017.11.054

[30] Ghanbari H, Mohammadi E, Fooeik AML, Kumar RR, Stauvermann PJ, Shabani M. Cryptocurrency portfolio allocation under credibilistic CVaR criterion and practical constraints. Risks. 2024;12(10):163.

[31] Tobin J. Liquidity preference as behavior towards risk. The Review of Economic Studies. 1958;25(2):65. doi: 10.2307/2296205

[32] Sharpe WF. A simplified model for portfolio analysis. Management Science. 1963;9(2):277–93. doi: 10.1287/mnsc.9.2.277

[33] Ritter LS, Renwick FB. Asset management and investor portfolio behavior: theory and practice. The Journal of Finance. 1969;24(2):181–206. doi: 10.1111/j.1540-6261.1969.tb01674.x

[34] Merton RC. Lifetime portfolio selection under uncertainty: The continuous-time case. The Review of Economics and Statistics. 1969;51(3):247–57. doi: 10.2307/1926560

[35] Elton EJ, Gruber MJ, Padberg MW. Simple Criteria for Optimal Portfolio Selection. The Journal of Finance. 1976;31(5):1341–57. doi: 10.2307/2326684

[36] Grauer RR, Hakansson NH. On the use of mean-variance and quadratic approximations in implementing dynamic investment strategies: A comparison of returns and investment policies. Management Science. 1993;39(7):856–71. doi: 10.1287/mnsc.39.7.856

[37] Chen Z, Knez PJ. Portfolio performance measurement: theory and applications. Rev Financ Stud. 1996;9(2):511–55. doi: 10.1093/rfs/9.2.511

[38] Abdelaziz FB, Aouni B, Fayedh RE. Multi-objective stochastic programming for portfolio selection. European Journal of Operational Research. 2007;177(3):1811–23. doi: 10.1016/j.ejor.2005.10.021

[39] Liu H, Loewenstein M. Optimal Portfolio Selection with Transaction Costs and Finite Horizons. Rev Financ Stud. 2002;15(3):805–35. doi: 10.1093/rfs/15.3.805

[40] Brown DB, Smith JE. Dynamic portfolio optimization with transaction costs: heuristics and dual bounds. Management Science. 2011;57(10):1752–70. doi: 10.1287/mnsc.1110.1377

[41] Tu J, Zhou G. Incorporating economic objectives into Bayesian priors: Portfolio choice under parameter uncertainty. Journal of Financial and Quantitative Analysis. 2010;45(4):959–86. doi: 10.1017/S0022109010000335

[42] Li J, Xu J. Multi-objective portfolio selection model with fuzzy random returns and a compromise approach-based genetic algorithm. Information Sciences. 2013;220:507–21. doi: 10.1016/j.ins.2012.07.005

[43] Yunusoglu MG, Selim H. A fuzzy rule based expert system for stock evaluation and portfolio construction: an application to Istanbul Stock Exchange. Expert Systems with Applications. 2013;40(3):908–20. doi: 10.1016/j.eswa.2012.05.047

[44] Almahdi S, Yang SY. An adaptive portfolio trading system: a risk-return portfolio optimization using recurrent reinforcement learning with expected maximum drawdown. Expert Systems with Applications. 2017;87:267–79. doi: 10.1016/j.eswa.2017.06.023

[45] Larni-Fooeik A, Sadjadi SJ, Mohammadi E. Stochastic portfolio optimization: a regret-based approach on volatility risk measures: An empirical evidence from The New York stock market. PLoS One. 2024;19(4):e0299699. doi: 10.1371/journal.pone.0299699 38648229 PMC11034657

[46] Kellerer H, Mansini R, Speranza MG. No title found. Annals of Operations Research. 2000;99(1/4):287–304. doi: 10.1023/A:1019279918596

[47] Cura T. Particle swarm optimization approach to portfolio optimization. Nonlinear Analysis-Real World Applications. 2009;10(4). doi: 10.1016/j.nonrwa.2008.04.023

[48] Rockafellar RT, Uryasev S. Optimization of conditional value-at-risk. JOR. 2000;2(3):21–41. doi: 10.21314/JOR.2000.038

[49] Liu Y-J, Zhang W-G, Xu W-J. Fuzzy multi-period portfolio selection optimization models using multiple criteria. Automatica. 2012;48(12):3042–53. doi: 10.1016/j.automatica.2012.08.036

[50] Liu Y-J, Zhang W-G, Zhang P. A multi-period portfolio selection optimization model by using interval analysis. Economic Modelling. 2013;33:113–9. doi: 10.1016/j.econmod.2013.03.006

[51] Gupta P, Mehlawat MK, Inuiguchi M, Chandra S. Fuzzy portfolio optimization: Advances in hybrid multi-criteria methodologies. Studies in Fuzziness and Soft Computing. Berlin, Heidelberg: Springer; 2014. doi: 10.1007/978-3-642-54652-5

[52] Liu Y-J, Zhang W-G. A multi-period fuzzy portfolio optimization model with minimum transaction lots. European Journal of Operational Research. 2015;242(3):933–41. doi: 10.1016/j.ejor.2014.10.061

[53] Vercher E, Bermúdez JD. Portfolio optimization using a credibility mean-absolute semi-deviation model. Expert Systems with Applications. 2015;42(20):7121–31. doi: 10.1016/j.eswa.2015.05.020

[54] Yao H, Li Z, Li D. Multi-period mean-variance portfolio selection with stochastic interest rate and uncontrollable liability. European Journal of Operational Research. 2016;252(3):837–51. doi: 10.1016/j.ejor.2016.01.049

[55] Mehlawat MK. Credibilistic mean-entropy models for multi-period portfolio selection with multi-choice aspiration levels. Information Sciences. 2016;345:9–26. doi: 10.1016/j.ins.2016.01.042

[56] Guo S, Yu L, Li X, Kar S. Fuzzy multi-period portfolio selection with different investment horizons. European Journal of Operational Research. 2016;254(3):1026–35. doi: 10.1016/j.ejor.2016.04.055

[57] Mei X, DeMiguel V, Nogales FJ. Multiperiod portfolio optimization with multiple risky assets and general transaction costs. Journal of Banking & Finance. 2016;69:108–20. doi: 10.1016/j.jbankfin.2016.04.002

[58] Vaezi F, Sadjadi SJ, Makui A. A portfolio selection model based on the knapsack problem under uncertainty. PLOS ONE. 2019;14(5):e0213652. doi: 10.1371/journal.pone.0213652 PMC6493714 31042709

[59] Ding R, Uryasev S. Drawdown beta and portfolio optimization. Quantitative Finance. 2022;22(7):1265–76. doi: 10.1080/14697688.2022.2037698

[60] Caçador SC, Godinho PMC, Dias JMPCM. A minimax regret portfolio model based on the investor’s utility loss. Oper Res Int J. 2022;22(1):449–84. doi: 10.1007/s12351-020-00550-0

[61] Kagrecha A, Nair J, Jagannathan K. Constrained regret minimization for multi-criterion multi-armed bandits. Mach Learn. 2023;112(2):431–58. doi: 10.1007/s10994-022-06291-9

[62] Nasukawa T, Yi J. Sentiment analysis: capturing favorability using natural language processing. In: Proceedings of the 2nd international conference on Knowledge capture, New York, NY, USA, 2003. 70–7. 10.1145/945645.945658

[63] Tetlock PC. Giving content to investor sentiment: the role of media in the stock market. The Journal of Finance. 2007;62(3):1139–68. doi: 10.1111/j.1540-6261.2007.01232.x

[64] Schmeling M. Investor sentiment and stock returns: Some international evidence. Journal of Empirical Finance. 2009;16(3):394–408. doi: 10.1016/j.jempfin.2009.01.002

[65] Ribeiro FN, Araújo M, Gonçalves P, Benevenuto F, Gonçalves MA. SentiBench - a benchmark comparison of state-of-the-practice sentiment analysis methods. 2016. doi: 10.48550/arXiv.1512.01818

[66] Hutto C, Gilbert E. VADER: a parsimonious rule-based model for sentiment analysis of social media text. ICWSM. 2014;8(1):216–25. doi: 10.1609/icwsm.v8i1.14550

[67] Vaswani A. Attention is All You Need.

[68] Devlin J, Chang M-W, Lee K, Toutanova K. BERT: Pre-training of Deep Bidirectional Transformers for Language Understanding. In: Proceedings of the 2019 Conference of the North American Chapter of the Association for Computational Linguistics: Human Language Technologies, NAACL-HLT 2019, Minneapolis, MN, USA, 2019. 4171–86. 10.18653/V1/N19-1423

[69] Sun C, Huang L, Qiu X. Utilizing BERT for Aspect-Based Sentiment Analysis via Constructing Auxiliary Sentence. 2019. doi: 10.48550/arXiv.1903.09588

[70] Xu H, Liu B, Shu L, Yu PS. BERT Post-Training for Review Reading Comprehension and Aspect-based Sentiment Analysis. 2019. doi: 10.48550/arXiv.1904.02232

[71] Fatouros G, Soldatos J, Kouroumali K, Makridis G, Kyriazis D. Transforming sentiment analysis in the financial domain with ChatGPT. Machine Learning with Applications. 2023;14:100508. doi: 10.1016/j.mlwa.2023.100508

[72] Ribeiro FN, Araújo M, Gonçalves P, Gonçalves MA, Benevenuto F. SentiBench - a benchmark comparison of state-of-the-practice sentiment analysis methods. EPJ Data Sci. 2016;5(1):23. doi: 10.1140/epjds/s13688-016-0085-1

[73] Araci D, Genç Z. Financial sentiment analysis with pre-trained language models. 2020.

[74] Hiew JZG, Huang X, Mou H, Li D, Wu Q, Xu Y. BERT-based Financial Sentiment Index and LSTM-based Stock Return Predictability. 2022. doi: 10.48550/arXiv.1906.09024

[75] Wu Y, Jin Z, Shi C, Liang P, Zhan T. Research on the Application of Deep Learning-based BERT Model in Sentiment Analysis. 2024. doi: 10.48550/arXiv.2403.08217

[76] Shen Y, Zhang PK. Financial sentiment analysis on news and reports using large language models and finbert. 2024. doi: 10.48550/arXiv.2410.01987

[77] jun Gu W, hao Zhong Y, zun Li S, song Wei C, ting Dong L, yue Wang Z, et al. Predicting Stock Prices with FinBERT-LSTM: Integrating News Sentiment Analysis. In: Proceedings of the 2024 8th International Conference on Cloud and Big Data Computing, 2024. 67–72. 10.1145/3694860.3694870

[78] Joseph K, Wintoki MB, Zhang Z. Forecasting abnormal stock returns and trading volume using investor sentiment: evidence from online search. International Journal of Forecasting. 2011;27(4):1116–27. doi: 10.1016/j.ijforecast.2010.11.001

[79] Bollen J, Mao H, Zeng X. Twitter mood predicts the stock market. Journal of Computational Science. 2011;2(1):1–8. doi: 10.1016/j.jocs.2010.12.007

[80] Preis T, Moat HS, Stanley HE. Quantifying trading behavior in financial markets using Google Trends. Sci Rep. 2013;3:1684. doi: 10.1038/srep01684 23619126 PMC3635219

[81] Malo P, Sinha A, Korhonen P, Wallenius J, Takala P. Good debt or bad debt: Detecting semantic orientations in economic texts. J Assoc Inf Sci Technol. 2014;65(4):782–96. doi: 10.1002/asi.23062

[82] Guo K, Sun Y, Qian X. Can investor sentiment be used to predict the stock price? Dynamic analysis based on China stock market. Physica A: Statistical Mechanics and its Applications. 2017;469:390–6. doi: 10.1016/j.physa.2016.11.114

[83] Yang S, Rosenfeld J, Makutonin J. Financial aspect-based sentiment analysis using deep representations. 2018. doi: 10.48550/arXiv.1808.07931

[84] Ko C-R, Chang H-T. LSTM-based sentiment analysis for stock price forecast. PeerJ Comput Sci. 2021;7:e408. doi: 10.7717/peerj-cs.408 33817050 PMC7959635

[85] Luo W, Gong D. Pre-trained large language models for financial sentiment analysis. 2024. doi: 10.48550/arXiv.2401.05215

[86] Dempster MAH, Leemans V. An automated FX trading system using adaptive reinforcement learning. Expert Systems with Applications. 2006;30(3):543–52. doi: 10.1016/j.eswa.2005.10.012

[87] Li H, Dagli CH, Enke D. Short-term stock market timing prediction under reinforcement learning schemes. 2007. 233–40. 10.1109/ADPRL.2007.368193

[88] Kara Y, Acar Boyacioglu M, Baykan ÖK. Predicting direction of stock price index movement using artificial neural networks and support vector machines: The sample of the Istanbul Stock Exchange. Expert Systems with Applications. 2011;38(5):5311–9. doi: 10.1016/j.eswa.2010.10.027

[89] Huang C-F. A hybrid stock selection model using genetic algorithms and support vector regression. Applied Soft Computing. 2012;12(2):807–18. doi: 10.1016/j.asoc.2011.10.009

[90] Wang W, Li W, Zhang N, Liu K. Portfolio formation with preselection using deep learning from long-term financial data. Expert Systems with Applications. 2020;143:113042. doi: 10.1016/j.eswa.2019.113042

[91] Paiva FD, Cardoso RTN, Hanaoka GP, Duarte WM. Decision-making for financial trading: a fusion approach of machine learning and portfolio selection. Expert Systems with Applications. 2019;115:635–55. doi: 10.1016/j.eswa.2018.08.003

[92] Żbikowski K. Using Volume Weighted Support Vector Machines with walk forward testing and feature selection for the purpose of creating stock trading strategy. Expert Systems with Applications. 2015;42(4):1797–805. doi: 10.1016/j.eswa.2014.10.001

[93] Zheng J, Xin D, Cheng Q, Tian M. The random forest model for analyzing and forecasting the us stock market in the context of smart finance. 2023.

[94] Chong E, Han C, Park FC. Deep learning networks for stock market analysis and prediction: methodology, data representations, and case studies. Expert Systems with Applications. 2017;83:187–205. doi: 10.1016/j.eswa.2017.04.030

[95] Seethalakshmi R. Analysis of stock market predictor variables using linear regression.

[96] Di Persio L, Honchar O. Recurrent Neural Networks Approach to the Financial Forecast of Google Assets. 2017. Accessed 2024 November 26.

[97] Singh DSK. Stock price prediction using LSTM on Indian share market. 2023;8(5).

[98] Bao W, Yue J, Rao Y. A deep learning framework for financial time series using stacked autoencoders and long-short term memory. PLoS One. 2017;12(7):e0180944. doi: 10.1371/journal.pone.0180944 28708865 PMC5510866

[99] Gülmez B. Stock price prediction with optimized deep LSTM network with artificial rabbits optimization algorithm. Expert Systems with Applications. 2023;227:120346. doi: 10.1016/j.eswa.2023.120346

[100] Pang X, Zhou Y, Wang P, Lin W, Chang V. An innovative neural network approach for stock market prediction. J Supercomput. 2020;76(3):2098–118. doi: 10.1007/s11227-017-2228-y

[101] Gil GL, Duhamel-Sebline P, McCarren A. An evaluation of deep learning models for stock market trend prediction. 2024. doi: 10.48550/arXiv.2408.12408

[102] Liu S, Zhang X, Wang Y, Feng G. Recurrent convolutional neural kernel model for stock price movement prediction. PLoS One. 2020;15(6):e0234206. doi: 10.1371/journal.pone.0234206 32530923 PMC7292408

[103] Qiu J, Wang B, Zhou C. Forecasting stock prices with long-short term memory neural network based on attention mechanism. PLoS One. 2020;15(1):e0227222. doi: 10.1371/journal.pone.0227222 31899770 PMC6941898

[104] Kao LJ, Chiu CC, Lu CJ, Yang JL. Integration of nonlinear independent component analysis and support vector regression for stock price forecasting. Neurocomput. 2013;99:534–42. doi: 10.1016/j.neucom.2012.06.037

[105] Rather AM, Agarwal A, Sastry VN. Recurrent neural network and a hybrid model for prediction of stock returns. Expert Systems with Applications. 2015;42(6):3234–41. doi: 10.1016/j.eswa.2014.12.003

[106] Kraus M, Feuerriegel S. Decision support from financial disclosures with deep neural networks and transfer learning. Decision Support Systems. 2017;104:38–48. doi: 10.1016/j.dss.2017.10.001

[107] Tsantekidis A, Passalis N, Tefas A, Kanniainen J, Gabbouj M, Iosifidis A. Forecasting Stock Prices from the Limit Order Book Using Convolutional Neural Networks. In: 2017 IEEE 19th Conference on Business Informatics (CBI), Thessaloniki, Greece, 2017. 7–12. 10.1109/CBI.2017.23

[108] Ta V-D, Liu C-M, Addis D. Prediction and Portfolio Optimization in Quantitative Trading Using Machine Learning Techniques. In: Proceedings of the Ninth International Symposium on Information and Communication Technology - SoICT 2018, 2018. 98–105. 10.1145/3287921.3287963

[109] Baek Y, Kim HY. ModAugNet: a new forecasting framework for stock market index value with an overfitting prevention LSTM module and a prediction LSTM module. Expert Systems with Applications. 2018;113:457–80. doi: 10.1016/j.eswa.2018.07.019

[110] Liu Y. Novel volatility forecasting using deep learning–long short term memory recurrent neural networks. Expert Systems with Applications. 2019;132:99–109. doi: 10.1016/j.eswa.2019.04.038

[111] Zhang Y, Yan B, Aasma MA. A novel deep learning framework: Prediction and analysis of financial time series using CEEMD and LSTM. Expert Systems with Applications. 2020;159:113609. doi: 10.1016/j.eswa.2020.113609

[112] Sun S, Wang S, Wei Y. A new ensemble deep learning approach for exchange rates forecasting and trading. Advanced Engineering Informatics. 2020;46:101160. doi: 10.1016/j.aei.2020.101160

[113] Lei K, Zhang B, Li Y, Yang M, Shen Y. Time-driven feature-aware jointly deep reinforcement learning for financial signal representation and algorithmic trading. Expert Syst Appl. 2020;140:112872. doi: 10.1016/j.eswa.2019.112872

[114] Chen W, Zhang H, Mehlawat MK, Jia L. Mean–variance portfolio optimization using machine learning-based stock price prediction. Appl Soft Comp. 2021;100:106943. doi: 10.1016/j.asoc.2020.106943

[115] Iyyappan M, Ahmad S, Jha S, Alam A, Yaseen M, Abdeljaber HAM. A novel AI-based stock market prediction using machine learning algorithm. Scientific Programming. 2022;2022(1):4808088. doi: 10.1155/2022/4808088

[116] Ma Y, Han R, Wang W. Prediction-based portfolio optimization models using deep neural networks. IEEE Access. 2020;8:115393–405. doi: 10.1109/ACCESS.2020.3003819

[117] Aldhyani THH, Alzahrani A. Framework for predicting and modeling stock market prices based on deep learning algorithms. Electronics. 2022;11(19):Art. no. 19. doi: 10.3390/electronics11193149

[118] Mukherjee S, Sadhukhan B, Sarkar N, Roy D, De S. Stock market prediction using deep learning algorithms. CAAI Trans Intelligence Technology. 2023;8(1):82–94. doi: 10.1049/cit2.12059

[119] Sarma SLVVD, Sekhar DV, Murali G. Stock market analysis with the usage of machine learning and deep learning algorithms. Bulletin EEI. 2023;12(1):552–60. doi: 10.11591/eei.v12i1.4305

[120] Chavhan S, Raj P, Raj P, Dutta AK, Rodrigues JJPC. Deep learning approaches for stock price prediction: a comparative study of LSTM, RNN, and GRU models. In: 2024 9th International Conference on Smart and Sustainable Technologies (SpliTech), 2024. 01–6. 10.23919/splitech61897.2024.10612666

[121] Gautam B, Kandel S, Shrestha M, Thakur S. Comparative analysis of machine learning models for stock price prediction: leveraging LSTM for real-time forecasting. Jf Computer and Communications. 2024;12(8):Art. no. 8. doi: 10.4236/jcc.2024.128004

[122] Behura JP, Pande SD, Ramesh JVN. Stock Price Prediction using Multi-Layered Sequential LSTM. EAI Endorsed Transactions on Scalable Information Systems. 2024;11(4):Art. no. 4. doi: 10.4108/eetsis.4585

[123] Furizal A, Ma’arif AA, Firdaus AA, Suwarno I. Capability of hybrid long short-term memory in stock price prediction: a comprehensive literature review. https://openurl.ebsco.com/contentitem/doi:10.31763%2Fijrcs.v4i3.1489?sid=ebsco:plink:crawler&id=ebsco:doi:10.31763%2Fijrcs.v4i3.1489

[124] Yu Y, Si X, Hu C, Zhang J. A review of recurrent neural networks: LSTM cells and network architectures. Neural Comput. 2019;31(7):1235–70. doi: 10.1162/neco_a_01199 31113301

[125] Santos AAP, Tessari C. Técnicas quantitativas de otimização de carteiras aplicadas ao mercado de ações brasileiro. Brazilian Review of Finance. 2012;10(3):Art. no. 3. doi: 10.12660/rbfin.v10n3.2012.3865

[126] Romero C. A general structure of achievement function for a goal programming model. European J Operat Res. 2004;153(3):675–86. doi: 10.1016/s0377-2217(02)00793-2

[127] Jones D, Tamiz M. Practical goal programming. International series in operations research & management science. Boston, MA: Springer US; 2010. doi: 10.1007/978-1-4419-5771-9

[128] Ignizio JP. A review of goal programming: a tool for multiobjective analysis. J Oper Res Soc. 1978;29(11):1109–19. doi: 10.1057/jors.1978.243

[129] Ballestero E, Garcia-Bernabeu A, Hilario A. Portfolio selection by goal programming techniques. In: Ballestero E, Pérez-Gladish B, Garcia-Bernabeu A, editors. Socially Responsible Investment. Cham: Springer International Publishing; 2015. 111–29. doi: 10.1007/978-3-319-11836-9_5

[130] Saaty TL. The analytic hierarchy process: planning, priority setting, resource allocation. New York; London: McGraw-Hill International Book Co.; 1980.

[131] Donegan HA, Dodd FJ, McMaster TBM. Royal Statistical Society Publications. doi: 10.2307/2348551

[132] Sharpe WF. The sharpe ratio. JPM. 1994;21(1):49–58. doi: 10.3905/jpm.1994.409501

[133] Atmaca ME. Portfolio management and performance improvement with Sharpe and Treynor ratios in electricity markets. Energy Reports. 2022;8:192–201. doi: 10.1016/j.egyr.2021.11.287

[134] Srivastava P, Mazhar SS. Comparative analysis of Sharpe and Sortino ratio with reference to top ten banking and finance sector mutual funds. International J Management Studies. 2018;V(4(2)):93. doi: 10.18843/ijms/v5i4(2)/10

[135] Colasanto F, Grilli L, Santoro D, Villani G. BERT’s sentiment score for portfolio optimization: a fine-tuned view in Black and Litterman model. Neural Comput Appl. 2022;34(20):17507–21. doi: 10.1007/s00521-022-07403-1 35669537 PMC9150638

[136] Leow EKW, Nguyen BP, Chua MCH. Robo-advisor using genetic algorithm and BERT sentiments from tweets for hybrid portfolio optimisation. Expert Systems with Applications. 2021;179:115060. doi: 10.1016/j.eswa.2021.115060

[137] Day M-Y, Lee C-C. Deep learning for financial sentiment analysis on finance news providers. In: 2016 IEEE/ACM International Conference on Advances in Social Networks Analysis and Mining (ASONAM), 2016. 1127–34. 10.1109/ASONAM.2016.7752381

[138] Fatouros G, Soldatos J, Kouroumali K, Makridis G, Kyriazis D. Transforming sentiment analysis in the financial domain with ChatGPT. Machine Learning with Applications. 2023;14:100508.

[139] Seshakagari HRB, Umashankar A, Harikala T, Jayasree L, Severance J. Dynamic financial sentiment analysis and market forecasting through large language models. International J Human Comput Intelligence. 2025;4(1):Art. no. 1. doi: 10.5281/zenodo.15111609

