# Supplementary material for: A multi-objective portfolio optimization model incorporating sentiment analysis of quarterly reports and LSTM-based price prediction
Source: PLoS One. 2026 Jul 9;21(7):e0335036. doi: 10.1371/journal.pone.0335036 (PMC13349311; doi:10.1371/journal.pone.0335036)
Supplement: Attachment — Submitted filename: Authors Response Files.docx [file pone.0335036.s002.docx]

**Original Manuscript ID:** PONE-D-25-02047

Original Article Title: " **A Multi-Objective Portfolio Optimization Model Incorporating Sentiment Analysis of Quarterly Reports and LSTM-based Price Prediction**"

**To:** Plos One Editor

**Re:** Response to reviewers

Dear Editor,

Thank you for giving us the opportunity to revise our manuscript and address the reviewers’ valuable comments. We sincerely appreciate your time and consideration, and we are grateful for the constructive feedback that has helped us improve the quality of our work.

We are uploading (a) our point-by-point response to the comments (below) (response to reviewers, under “Author’s Response Files*”*), (b) an updated manuscript with yellow highlighting indicating changes (as “Highlighted PDF*”*), and (c) a clean updated manuscript without highlights (“Main Manuscript”*).*

Best regards,

Seyed Jafar Sadjadi.

**Comment # 1:** The manuscript is well-structured and presents a novel approach to portfolio optimization by integrating sentiment analysis and LSTM-based price prediction. However, the transitions between sections could be smoother to enhance readability.

**Author response:** Thank you for your insightful feedback. We have thoroughly reviewed the manuscript, corrected the way the text is expressed, and made other technical edits such as fonts and paragraphing.

**Author action:** We have conducted a comprehensive and thorough review of the entire manuscript and, in accordance with the reviewers' comments, have made the following changes or improvements:

- Improved the text in expressing the relationship between different parts of the article
- Improved the quality of the images in the article
- Improved the quality of the literature review tables in the manuscript (Tables 1, 2, and 3)
- Corrected some typographical errors

***The mentioned changes have been highlighted in yellow in the entire manuscript***

**Comment # 2:** The authors should provide more detailed information on the preprocessing of the quarterly report texts used in the sentiment analysis. Additionally, the configuration of the LSTM model, including the rationale behind the chosen architecture and hyperparameters, needs to be explained。.

**Author response:** Thank you for your valuable feedback.

1. *Detailed information on the preprocessing of the quarterly report texts used in the sentiment analysis:* The process of extracting the score for company future outlooks is as follows: first, companies’ quarterly reports are extracted, and then insights and information from these reports are extracted using the Sider AI tool. Table 4 shows two examples of extracted Outlooks. The extracted outlooks are then fed to the FinBERT model and sentiment analysis is performed on them, and then the FinBERT model outputs are converted into $\gamma_{i}$ scores using the AHP method. We also added **Figure 5** to the article to clarify the process of extracting companies' Outlook scores from quarterly reports. In addition, two of the outlooks (future trends) extracted with the help of the Sider artificial intelligence tool are shown as examples in Table 4, and the complete data has been uploaded to GitHub and its link is as follows.

*<https://github.com/esipour93/DJIA_Textual_Data-1.git>*

2. *The configuration of the LSTM model:* LSTM networks were first introduced by Hochreiter and Schmidhuber [24] as a technique for capturing and learning sequential patterns. LSTM networks are a specialized form RNNs that are capable of retaining information over extended periods, making them more effective than traditional RNNs in handling long-term dependencies [92]. Graves and Schmidhuber [126] provide evidence that LSTM networks are capable of overcoming the inherent issues that were previously present and memorizing temporal patterns over an extended length of time. Consequently, in our study, we will make use of this model to forecast the prices of stocks in the future. LSTM networks are composed of an input layer, several hidden layers, and an output layer. What distinguishes LSTMs from other types of networks is the inclusion of memory cells within the hidden layers. Figure 4 shows the architecture of an LSTM memory cell, which plays a crucial role in storing and managing information over time. To effectively model time-series data such as stock prices, the LSTM model is configured with the following components:
1. *Input Layer:* The input layer takes in a sequence of historical financial data, such as the opening price, closing price, high, low, and trading volume. This input is typically structured into time windows (e.g., 60-time steps) to give the model temporal context. Each input vector at a time step can include multiple features.
2. *LSTM Layers:* The core of the model is the LSTM layer (or layers), which consists of memory blocks called cells. *Each* cell maintains an internal state, controlled by three gates:

**• Forget Gate (fₜ):** Decides what information to discard from the cell state.

• **Input Gate (iₜ):** Determines which new information to store in the cell state.

• **Output Gate (oₜ):** Controls how much of the internal state to pass to the output.

The input and hidden states are represented by $x_{t}$ and $h_{t}$ at time t. respectively, $s_{t}$ is adjusting its cell state. The input gate controls which data should be stored in the memory cell, the output gate governs what information is extracted from the memory cell, and the forget gate determines which data should be discarded. The mathematical formulation of an LSTM cell is as follows:

$$f_{t}=sigmoid\left( W_{f,x}x_{t}+W_{f,h}h_{t-1}+b_{f} \right)$$

(1)

$$\tilde{s_{t}}=sigmoid\left( W_{\tilde{s},x}x_{t}+W_{\tilde{s},h}h_{t-1}+b_{\tilde{s_{t}}} \right)$$

(2)

$$i_{t}=sigmoid\left( W_{i,x}x_{t}+W_{i,h}h_{t-1}+b_{i} \right)$$

(3)

$$s_{t}=f_{t}*s_{t-1}+i_{t}*\tilde{s_{t}}$$

(4)

$$o_{t}=sigmoid\left( W_{o,x}x_{t}+W_{o,h}h_{t-1}+b_{o} \right)$$

(5)

$$h_{t}=o_{t}*tanh(s_{t})$$

(6)

Where $W_{f,x}$,$W_{f,h}$,$W_{\tilde{s},x}$,$W_{\tilde{s},h}$,$W_{i,x}$,$W_{i,h}$,$W_{o,x}$and $W_{o,h}$are weight matrices, $b_{f}$, $b_{\tilde{s}}$, $b_{i}$, and $b_{o}$ are the bias vectors associated with each of the respective gates. The bias vectors are incorporated to enhance the model's adaptability to the data. The bias vectors, $b_{\tilde{s}}$, $b_{i}$, and $b_{o}$ are initialized to zero, whereas the bias $b_{f}$ the forget gate in the LSTM is initialized to 1.0. The symbol ∗ represents element-wise multiplication. These operations allow the model to regulate information flow dynamically and effectively manage both short-term and long-term dependencies.

3. *Stacked LSTM Architecture:* In many advanced configurations, multiple LSTM layers are stacked to increase the model’s representational power. The output of each LSTM layer is passed to the next layer, enabling deeper abstraction of sequential features.
4. *Dropout Regularization:* Dropout layers are inserted between LSTM layers to prevent overfitting by randomly deactivating a fraction of the neurons during training. This improves generalization, especially when the dataset is small or noisy.
5. *Dense (Fully Connected) Layer:* The final dense layer maps the high-level sequence representations to a single output value (e.g., predicted stock price). For regression tasks, the activation function is typically linear.
6. *Compilation and Training:* The model is compiled using a loss function such as Mean Squared Error (MSE), which is appropriate for continuous output variables. The optimizer is often Adam, which adapts learning rates and accelerates convergence. Training is conducted over multiple epochs, with early stopping or validation monitoring to prevent overfitting.

The architecture of the LSTM network is particularly advantageous for modeling financial time series data, where both short-term fluctuations and long-range temporal dependencies play a critical role in shaping future trends. In the context of stock market forecasting, price movements are rarely independent of historical behavior; rather, they are influenced by recurring patterns, investor sentiment, and macroeconomic events that unfold over varying timescales. LSTM networks are inherently designed to capture such temporal structures by maintaining a memory of past states while dynamically adjusting their focus to recent changes. This ability allows them to outperform traditional machine learning models, which typically rely on fixed-size input vectors and handcrafted features. Unlike methods such as linear regression or support vector machines that require domain-specific feature engineering to capture time dependencies, LSTM models automatically learn relevant temporal representations directly from raw sequential data. This makes them not only more adaptable to the complex dynamics of financial markets but also more scalable for real-world forecasting applications where patterns may shift over time. For more information, please refer to the article by Yu et al. [124].

Article DOI: <https://doi.org/10.1162/neco_a_01199>

3. *Hyperparameters:* The hyperparameters of the LSTM model, including the number of layers, the number of units per layer, dropout rates, batch size, and learning rate, were determined through a manual trial-and-error approach. Various configurations were tested based on values commonly used in related literature and prior experience with time-series forecasting tasks. The performance of each configuration was evaluated using validation data and standard metrics such as RMSE and MAE. The final set of hyperparameters was selected based on the best overall performance, balancing accuracy, model complexity, and overfitting prevention.

**Author action:** We have added the points made in the text of the article clearly and with full explanations.

***The mentioned changes have been highlighted in yellow in Section References.***

**Comment # 3**: The weighting scheme used in the weighted goal programming (WGP) method is not well justified.

**Author response:** We appreciate the reviewer’s comment regarding the justification of the weighting scheme used in the Weighted Goal Programming (WGP) model. To address this, we clarify that the Analytic Hierarchy Process (AHP) was selected as the weighting method due to its well-established ability to systematically capture expert judgments in multi-criteria decision-making problems. AHP is particularly suitable for contexts where multiple, often conflicting goals such as return, risk, and outlook must be prioritized based on subjective preferences or strategic considerations. Through pairwise comparisons and consistency checks, AHP provides a transparent and consistent way of deriving weights that reflect the relative importance of the goals from a decision-maker’s perspective. In this study, we collected expert judgments (or stakeholder inputs) to evaluate the relative importance of the three goals. These inputs were then processed using the AHP methodology to derive normalized weights, which were subsequently used in the WGP model. The use of AHP not only ensures methodological rigor in weight derivation but also allows for the integration of qualitative judgments in a quantitative optimization framework.Therefore, the weighting scheme is justified as it is derived from a structured decision-making process that is widely validated in the literature and appropriate for the problem context. The process begins with defining the objective, identifying criteria and subcriteria, and constructing a hierarchy. Next, pairwise comparisons between criteria are made using a relative importance scale, which typically ranges from 1 (equal importance) to 9 (extreme importance). These comparisons are used to create a pairwise comparison matrix from which the weights of the criteria are calculated - often by normalizing the matrix and calculating the average of each row. Finally, a consistency ratio is calculated to ensure that the judgments are consistent. If this ratio is acceptable (usually less than 0.1), the resulting weights can be used in the decision-making process. Here, we weighted the three objectives according to expert opinion and formed optimal portfolios based on these results.

**Author action:** We updated the manuscript by adding these description.

***The above description added to manuscript and highlighted in yellow.***

**Comment # 4:** While the integration of sentiment analysis and LSTM for portfolio optimization is innovative, the authors should more explicitly compare their approach with traditional portfolio optimization methods to highlight the added value of their model.

**Author response:** Thank you for your valuable comment. We agree that comparing our proposed model with traditional portfolio optimization methods is essential to demonstrate its added value. Accordingly, we have added a new subsection titled **“****Comparison with Other Portfolio Optimization Model”** to Section 6.

In this paper, we tried to present a new model in which we used new neural network models and artificial intelligence tools. In this model, we tried to add a new criterion as the outlook of the possible future price movement to improve the model results. Our proposed model included two types of text and price data, which we calculated the price data using the LSTM model, and for text data, we collected the quarterly reports of DJIA companies from the Yahoo Finance website and extracted the companies' outlook for the next quarter using the Cider artificial intelligence tool. To convert this text data into a score, we used the FinBERT model and performed sentiment analysis on the outlooks, and considering that the output of the FinBERT model was a vector (positive, neutral, negative), we used the AHP method to convert this vector into a score and finally extracted the outlook score, this process can be seen in Figure 5. In fact, we tried to present a State-of-the-Art model. The topic of sentiment analysis in most previous models has been that first, sentiment analysis was performed on Twitter data, websites, and social networks, and second, the results were used as one of the features for price prediction. While in our study, we have used data from companies' quarterly reports with an innovative approach. Among the previous studies, the following can be mentioned:

Colasanto et al. [135] In one study, sentiment scores, particularly polarity values, were incorporated into stock price forecasting by introducing them as an additional “view” in the Black-Letterman framework. These scores were extracted from textual analysis of Financial Times articles and were associated with various events, both favorable and unfavorable, that affected specific stocks. The key distinction of the present study is in the application of the FinBERT model, which is used not only as a predictive feature but also to extract insights into future performance from corporate disclosures. Unlike previous approaches that used sentiment as a direct input for price forecasting, our method uses FinBERT to assess the outlook of companies, thereby informing investment decisions with a more interpretative and qualitative layer. Leow et al. [136] introduced two novel models SAW and SMPT which incorporate real-time market sentiment derived from Twitter data, analyzed using BERT. These models adjust portfolio allocation weights based on the extracted sentiment signals. To optimize their performance, genetic algorithms are employed with objectives such as maximizing cumulative returns and minimizing volatility. In contrast, the present study relies on sentiment extracted from corporate quarterly reports to assess future outlooks, offering a more fundamental and document-based approach to sentiment analysis. Day and Li [137] examined the influence of various financial information sources on investment decisions and explored how deep learning techniques can enhance the accuracy of financial news classification. Their empirical findings demonstrated that the type of financial source significantly affects investor behavior and decision-making outcomes, and that classification performance benefits from the application of deep learning models. However, unlike the current study, their research did not address portfolio optimization; instead, it focused solely on analyzing the effects of information quality and classification on investment behavior. Fatouros et al. [138] conducted a pioneering study exploring the capabilities of large language models specifically ChatGPT-3.5 in the context of financial sentiment analysis, with a focus on the FOREX market. Employing a goal-free prompting strategy, they evaluated various ChatGPT prompts on a substantial dataset of FOREX related news headlines. Model performance was assessed using standard evaluation metrics, including precision, recall, F1-score, and MAE for sentiment classification. Notably, the study does not propose a structured or model-driven investment framework; rather, it centers on assessing the viability of LLMs for sentiment detection. Seshakagari et al. [139] employed three advanced models FinBERT, GPT-4, and T5 for the task of financial sentiment classification. By evaluating these models using precision, recall, and F1 score, the study contributes to the field by offering comparative insights that guide the selection of the most suitable model depending on specific application needs in financial sentiment analysis. However, similar to other works in this domain, their study does not extend to portfolio construction or optimization, focusing solely on the classification task.

Generally, the proposed model distinguishes itself through two notable and innovative features. First, it integrates qualitative news data extracted from companies’ quarterly reports, allowing the model to capture sentiment, forward-looking statements, and performance insights that are not typically reflected in historical price data alone. This incorporation of textual and contextual information enhances the depth of the analysis and supports more informed investment decisions. Second, the model introduces a three-objective optimization framework, expanding beyond the traditional focus on return maximization and risk minimization. The third objective specifically evaluates the future outlook of companies, derived from financial reports and qualitative assessments. This added dimension allows the model to account for anticipated growth, market positioning, and management guidance factors that are increasingly relevant in dynamic and uncertain market environments. The effectiveness of this approach is demonstrated in Table 12, which presents the performance metrics of the optimized portfolios. Notably, the Sharpe ratios of portfolios that assign greater weight to the third objective the forward-looking outlook show superior risk-adjusted returns. This indicates that incorporating qualitative future expectations alongside quantitative financial data can lead to more robust and strategically balanced investment outcomes.

**Author action:** The manuscript has been revised and updated with additional details to enhance the clarity, precision, and depth of the proposed approach. These revisions ensure that key concepts are explained more fully, improve the overall coherence of the content, and address potential gaps in understanding. A comprehensive comparison of the proposed model with previous studies is provided.

***The above changes have been highlighted in yellow in the section 6.***

**Comment # 5:** The study's reliance on data from the DJIA companies limits the generalizability of the findings. The authors should consider expanding the dataset to include a broader range of stocks from different markets to validate the model's robustness.

**Author response:** We sincerely appreciate your valuable feedback. We fully agree that relying solely on data from DJIA companies may limit the generalizability of our findings. The selection of the Dow Jones Industrial Average (DJIA) companies as the focus of this case study is based on several compelling reasons related to data quality, market representativeness, and research robustness.

1. *Representation of market leaders:*

The DJIA consists of 30 large, well-established public companies that are leaders in their respective industries. These companies are widely recognized as pioneers in the U.S. economy and financial markets. By focusing on these companies, this study captures the trends and dynamics that significantly influence overall market movements.

2. *Data availability and reliability:*

Due to their prominence, DJIA companies have extensive, high-quality, and well-documented historical data available. This includes stock prices, financial statements, and market indices with minimal missing or inconsistent values. Such reliable data is critical for building robust and valid financial models.

3. *Market stability and liquidity:*

The DJIA components are characterized by large market capitalization and high liquidity. This reduces noise from illiquid stocks or extreme volatility and facilitates a clearer analysis of market behavior and model performance.

4. *Well-known benchmark:*

The DJIA index is one of the oldest and most popular stock market indices globally, making findings based on its components highly relevant and interpretable by academics, practitioners, and policymakers. This relevance enhances the practical implications of the study.

5. *Controlled complexity:*

By limiting the scope to a well-defined and relatively stable group of companies, this study controls for heterogeneity resulting from the composition of companies from diverse markets or sectors with different regulatory and economic environments. This allows for a more focused assessment of the capabilities of the proposed model.

6. *Essential Step for Broader Research:*

While the DJIA does not cover the entire market spectrum, it serves as a meaningful and manageable starting point. The insights gained here provide a solid foundation before extending the analysis to a more diverse and broader dataset in future research.

**In summary,** the selection of DJIA companies balances data availability, market representativeness, and analytical rigor, making it a strategic and appropriate choice for this case study. However, we agree that expanding the dataset to include a wider range of stocks from different markets would enhance the robustness and applicability of the model. Accordingly, this is explicitly mentioned as an avenue for future research in the Discussion section. Future studies will aim to validate and extend the model on more diverse datasets to improve its generalizability. This point will also be mentioned in the Limitations section.

**Author action:** Explanations related to data access restrictions were added to the end of the article, and suggestions for future studies were added in the results section (Section 7).

***The above changes have been highlighted in yellow in the section 7.***

Once again, we thank the reviewer for this insightful comment, which will undoubtedly help improve the quality of our work. Finaly we hope these revisions sufficiently address your concern.
