# Supplementary material for: A multi-objective portfolio optimization model incorporating sentiment analysis of quarterly reports and LSTM-based price prediction
Source: PLoS One. 2026 Jul 9;21(7):e0335036. doi: 10.1371/journal.pone.0335036 (PMC13349311; doi:10.1371/journal.pone.0335036)
Supplement: Peer review history

# Peer review: Decision Letter 0

**DOI:** 10.1371/journal.pone.0335036.r001

**Authors:** Junhuan Zhang

5 May 2025

PONE-D-25-02047A Multi-Objective Portfolio Optimization Model Incorporating Sentiment Analysis of Quarterly Reports and LSTM-based Price PredictionPLOS ONE

Dear Dr. Sadjadi,

Thank you for submitting your manuscript to PLOS ONE. After careful consideration, we feel that it has merit but does not fully meet PLOS ONE’s publication criteria as it currently stands. Therefore, we invite you to submit a revised version of the manuscript that addresses the points raised during the review process.

Please submit your revised manuscript by Jun 19 2025 11:59PM. If you will need more time than this to complete your revisions, please reply to this message or contact the journal office at plosone@plos.org. When you're ready to submit your revision, log on to https://www.editorialmanager.com/pone/ and select the 'Submissions Needing Revision' folder to locate your manuscript file.

Please include the following items when submitting your revised manuscript:

- A rebuttal letter that responds to each point raised by the academic editor and reviewer(s). You should upload this letter as a separate file labeled 'Response to Reviewers'.
- A marked-up copy of your manuscript that highlights changes made to the original version. You should upload this as a separate file labeled 'Revised Manuscript with Track Changes'.
- An unmarked version of your revised paper without tracked changes. You should upload this as a separate file labeled 'Manuscript'.

If you would like to make changes to your financial disclosure, please include your updated statement in your cover letter. Guidelines for resubmitting your figure files are available below the reviewer comments at the end of this letter.

If applicable, we recommend that you deposit your laboratory protocols in protocols.io to enhance the reproducibility of your results. Protocols.io assigns your protocol its own identifier (DOI) so that it can be cited independently in the future. For instructions see: https://journals.plos.org/plosone/s/submission-guidelines#loc-laboratory-protocols. Additionally, PLOS ONE offers an option for publishing peer-reviewed Lab Protocol articles, which describe protocols hosted on protocols.io. Read more information on sharing protocols at https://plos.org/protocols?utm_medium=editorial-email&utm_source=authorletters&utm_campaign=protocols.

We look forward to receiving your revised manuscript.

Kind regards,

Junhuan Zhang, PhD

Academic Editor

PLOS ONE

Journal Requirements:

When submitting your revision, we need you to address these additional requirements.

1.Please ensure that your manuscript meets PLOS ONE's style requirements, including those for file naming. The PLOS ONE style templates can be found at

https://journals.plos.org/plosone/s/file?id=wjVg/PLOSOne_formatting_sample_main_body.pdf and

https://journals.plos.org/plosone/s/file?id=ba62/PLOSOne_formatting_sample_title_authors_affiliations.pdf

2. Please note that PLOS ONE has specific guidelines on code sharing for submissions in which author-generated code underpins the findings in the manuscript. In these cases, we expect all author-generated code to be made available without restrictions upon publication of the work. Please review our guidelines at https://journals.plos.org/plosone/s/materials-and-software-sharing#loc-sharing-code and ensure that your code is shared in a way that follows best practice and facilitates reproducibility and reuse.

3. Please note that your Data Availability Statement is currently missing the repository name and/or the DOI/accession number of each dataset OR a direct link to access each database. If your manuscript is accepted for publication, you will be asked to provide these details on a very short timeline. We therefore suggest that you provide this information now, though we will not hold up the peer review process if you are unable.

4. Please ensure that you include a title page within your main document. You should list all authors and all affiliations as per our author instructions and clearly indicate the corresponding author.

[Note: HTML markup is below. Please do not edit.]

Reviewers' comments:

Reviewer's Responses to Questions

**Comments to the Author**

1. Is the manuscript technically sound, and do the data support the conclusions?

The manuscript must describe a technically sound piece of scientific research with data that supports the conclusions. Experiments must have been conducted rigorously, with appropriate controls, replication, and sample sizes. The conclusions must be drawn appropriately based on the data presented.

Reviewer #1: Partly

**********

2. Has the statistical analysis been performed appropriately and rigorously?

Reviewer #1: Yes

**********

3. Have the authors made all data underlying the findings in their manuscript fully available?

The PLOS Data policy requires authors to make all data underlying the findings described in their manuscript fully available without restriction, with rare exception (please refer to the Data Availability Statement in the manuscript PDF file). The data should be provided as part of the manuscript or its supporting information, or deposited to a public repository. For example, in addition to summary statistics, the data points behind means, medians and variance measures should be available. If there are restrictions on publicly sharing data—e.g. participant privacy or use of data from a third party—those must be specified.

Reviewer #1: No

**********

4. Is the manuscript presented in an intelligible fashion and written in standard English?

PLOS ONE does not copyedit accepted manuscripts, so the language in submitted articles must be clear, correct, and unambiguous. Any typographical or grammatical errors should be corrected at revision, so please note any specific errors here.

Reviewer #1: Yes

**********

5. Review Comments to the Author

Please use the space provided to explain your answers to the questions above. You may also include additional comments for the author, including concerns about dual publication, research ethics, or publication ethics. (Please upload your review as an attachment if it exceeds 20,000 characters)

Reviewer #1: This paper proposes a three-stage portfolio optimization model that combines sentiment analysis of quarterly reports with LSTM-based price prediction to enhance investment decisions. The model integrates risk, return, and sentiment trends, demonstrating high accuracy with DJIA data.

1. The manuscript is well-structured and presents a novel approach to portfolio optimization by integrating sentiment analysis and LSTM-based price prediction. However, the transitions between sections could be smoother to enhance readability.

2. The authors should provide more detailed information on the preprocessing of the quarterly report texts used in the sentiment analysis. Additionally, the configuration of the LSTM model, including the rationale behind the chosen architecture and hyperparameters, needs to be explained。

3. The weighting scheme used in the weighted goal programming (WGP) method is not well justified.

4. While the integration of sentiment analysis and LSTM for portfolio optimization is innovative, the authors should more explicitly compare their approach with traditional portfolio optimization methods to highlight the added value of their model.

5. The study's reliance on data from the DJIA companies limits the generalizability of the findings. The authors should consider expanding the dataset to include a broader range of stocks from different markets to validate the model's robustness.

**********

6. PLOS authors have the option to publish the peer review history of their article (what does this mean?). If published, this will include your full peer review and any attached files.

If you choose “no”, your identity will remain anonymous but your review may still be made public.

**Do you want your identity to be public for this peer review?**  For information about this choice, including consent withdrawal, please see our Privacy Policy.

Reviewer #1: No

**********

[NOTE: If reviewer comments were submitted as an attachment file, they will be attached to this email and accessible via the submission site. Please log into your account, locate the manuscript record, and check for the action link "View Attachments". If this link does not appear, there are no attachment files.]

While revising your submission, please upload your figure files to the Preflight Analysis and Conversion Engine (PACE) digital diagnostic tool, https://pacev2.apexcovantage.com/. PACE helps ensure that figures meet PLOS requirements. To use PACE, you must first register as a user. Registration is free. Then, login and navigate to the UPLOAD tab, where you will find detailed instructions on how to use the tool. If you encounter any issues or have any questions when using PACE, please email PLOS at figures@plos.org. Please note that Supporting Information files do not need this step.

# Author comment: Author response to Decision Letter 1

**DOI:** 10.1371/journal.pone.0335036.r002

19 May 2025

Original Manuscript ID: PONE-D-25-02047

Original Article Title: " A Multi-Objective Portfolio Optimization Model Incorporating Sentiment Analysis of Quarterly Reports and LSTM-based Price Prediction"

To: Plos One Editor

Re: Response to reviewers

Dear Editor,

Thank you for giving us the opportunity to revise our manuscript and address the reviewers’ valuable comments. We sincerely appreciate your time and consideration, and we are grateful for the constructive feedback that has helped us improve the quality of our work.

We are uploading (a) our point-by-point response to the comments (below) (response to reviewers, under “Author’s Response Files”), (b) an updated manuscript with yellow highlighting indicating changes (as “Highlighted PDF”), and (c) a clean updated manuscript without highlights (“Main Manuscript”).

Best regards,

Seyed Jafar Sadjadi.

Comment # 1: The manuscript is well-structured and presents a novel approach to portfolio optimization by integrating sentiment analysis and LSTM-based price prediction. However, the transitions between sections could be smoother to enhance readability.

Author response: Thank you for your insightful feedback. We have thoroughly reviewed the manuscript, corrected the way the text is expressed, and made other technical edits such as fonts and paragraphing.

Author action: We have conducted a comprehensive and thorough review of the entire manuscript and, in accordance with the reviewers' comments, have made the following changes or improvements:

Improved the text in expressing the relationship between different parts of the article

Improved the quality of the images in the article

Improved the quality of the literature review tables in the manuscript (Tables 1, 2, and 3)

Corrected some typographical errors

The mentioned changes have been highlighted in yellow in the entire manuscript

________________________________________

Comment # 2: The authors should provide more detailed information on the preprocessing of the quarterly report texts used in the sentiment analysis. Additionally, the configuration of the LSTM model, including the rationale behind the chosen architecture and hyperparameters, needs to be explained。.

Author response: Thank you for your valuable feedback.

Detailed information on the preprocessing of the quarterly report texts used in the sentiment analysis: The process of extracting the score for company future outlooks is as follows: first, companies’ quarterly reports are extracted, and then insights and information from these reports are extracted using the Sider AI tool. Table 4 shows two examples of extracted Outlooks. The extracted outlooks are then fed to the FinBERT model and sentiment analysis is performed on them, and then the FinBERT model outputs are converted into γ_i scores using the AHP method. We also added Figure 5 to the article to clarify the process of extracting companies' Outlook scores from quarterly reports. In addition, two of the outlooks (future trends) extracted with the help of the Sider artificial intelligence tool are shown as examples in Table 4, and the complete data has been uploaded to GitHub and its link is as follows.

https://github.com/esipour93/DJIA_Textual_Data-1.git

The configuration of the LSTM model: LSTM networks were first introduced by Hochreiter and Schmidhuber [24] as a technique for capturing and learning sequential patterns. LSTM networks are a specialized form RNNs that are capable of retaining information over extended periods, making them more effective than traditional RNNs in handling long-term dependencies [92]. Graves and Schmidhuber [126] provide evidence that LSTM networks are capable of overcoming the inherent issues that were previously present and memorizing temporal patterns over an extended length of time. Consequently, in our study, we will make use of this model to forecast the prices of stocks in the future. LSTM networks are composed of an input layer, several hidden layers, and an output layer. What distinguishes LSTMs from other types of networks is the inclusion of memory cells within the hidden layers. Figure 4 shows the architecture of an LSTM memory cell, which plays a crucial role in storing and managing information over time. To effectively model time-series data such as stock prices, the LSTM model is configured with the following components:

Input Layer: The input layer takes in a sequence of historical financial data, such as the opening price, closing price, high, low, and trading volume. This input is typically structured into time windows (e.g., 60-time steps) to give the model temporal context. Each input vector at a time step can include multiple features.

LSTM Layers: The core of the model is the LSTM layer (or layers), which consists of memory blocks called cells. Each cell maintains an internal state, controlled by three gates:

Forget Gate (fₜ): Decides what information to discard from the cell state.

Input Gate (iₜ): Determines which new information to store in the cell state.

Output Gate (oₜ): Controls how much of the internal state to pass to the output.

The input and hidden states are represented by x_t and h_t at time t. respectively, s_t is adjusting its cell state. The input gate controls which data should be stored in the memory cell, the output gate governs what information is extracted from the memory cell, and the forget gate determines which data should be discarded. The mathematical formulation of an LSTM cell is as follows:

f_t=sigmoid(W_(f,x) x_t+W_(f,h) h_(t-1)+b_f ) (1)

(s_t ) ~=sigmoid(W_(s ~,x) x_t+W_(s ~,h) h_(t-1)+b_(s_t ) ~ ) (2)

i_t=sigmoid(W_(i,x) x_t+W_(i,h) h_(t-1)+b_i ) (3)

s_t=f_t*s_(t-1)+i_t*(s_t ) ~ (4)

o_t=sigmoid(W_(o,x) x_t+W_(o,h) h_(t-1)+b_o ) (5)

h_t=o_t*tanh(s_t) (6)

Where W_(f,x), W_(f,h), W_(s ~,x), W_(s ~,h), W_(i,x), W_(i,h), W_(o,x)and W_(o,h)are weight matrices, b_f, b_s ~ , b_i, and b_o are the bias vectors associated with each of the respective gates. The bias vectors are incorporated to enhance the model's adaptability to the data. The bias vectors, b_s ~ , b_i, and b_o are initialized to zero, whereas the bias b_f the forget gate in the LSTM is initialized to 1.0. The symbol ∗ represents element-wise multiplication. These operations allow the model to regulate information flow dynamically and effectively manage both short-term and long-term dependencies.

Stacked LSTM Architecture: In many advanced configurations, multiple LSTM layers are stacked to increase the model’s representational power. The output of each LSTM layer is passed to the next layer, enabling deeper abstraction of sequential features.

Dropout Regularization: Dropout layers are inserted between LSTM layers to prevent overfitting by randomly deactivating a fraction of the neurons during training. This improves generalization, especially when the dataset is small or noisy.

Dense (Fully Connected) Layer: The final dense layer maps the high-level sequence representations to a single output value (e.g., predicted stock price). For regression tasks, the activation function is typically linear.

Compilation and Training: The model is compiled using a loss function such as Mean Squared Error (MSE), which is appropriate for continuous output variables. The optimizer is often Adam, which adapts learning rates and accelerates convergence. Training is conducted over multiple epochs, with early stopping or validation monitoring to prevent overfitting.

The architecture of the LSTM network is particularly advantageous for modeling financial time series data, where both short-term fluctuations and long-range temporal dependencies play a critical role in shaping future trends. In the context of stock market forecasting, price movements are rarely independent of historical behavior; rather, they are influenced by recurring patterns, investor sentiment, and macroeconomic events that unfold over varying timescales. LSTM networks are inherently designed to capture such temporal structures by maintaining a memory of past states while dynamically adjusting their focus to recent changes. This ability allows them to outperform traditional machine learning models, which typically rely on fixed-size input vectors and handcrafted features. Unlike methods such as linear regression or support vector machines that require domain-specific feature engineering to capture time dependencies, LSTM models automatically learn relevant temporal representations directly from raw sequential data. This makes them not only more adaptable to the complex dynamics of financial markets but also more scalable for real-world forecasting applications where patterns may shift over time. For more information, please refer to the article by Yu et al. [124].

Article DOI: https://doi.org/10.1162/neco_a_01199

Hyperparameters: The hyperparameters of the LSTM model, including the number of layers, the number of units per layer, dropout rates, batch size, and learning rate, were determined through a manual trial-and-error approach. Various configurations were tested based on values commonly used in related literature and prior experience with time-series forecasting tasks. The performance of each configuration was evaluated using validation data and standard metrics such as RMSE and MAE. The final set of hyperparameters was selected based on the best overall performance, balancing accuracy, model complexity, and overfitting prevention.

Author action: We have added the points made in the text of the article clearly and with full explanations.

The mentioned changes have been highlighted in yellow in Section References.

________________________________________

Comment # 3: The weighting scheme used in the weighted goal programming (WGP) method is not well justified.

Author response: We appreciate the reviewer’s comment regarding the justification of the weighting scheme used in the Weighted Goal Programming (WGP) model. To address this, we clarify that the Analytic Hierarchy Process (AHP) was selected as the weighting method due to its well-established ability to systematically capture expert judgments in multi-criteria decision-making problems. AHP is particularly suitable for contexts where multiple, often conflicting goals such as return, risk, and outlook must be prioritized based on subjective preferences or strategic considerations. Through pairwise comparisons and consistency checks, AHP provides a transparent and consistent way of deriving weights that reflect the relative importance of the goals from a decision-maker’s perspective. In this study, we collected expert judgments (or stakeholder inputs) to evaluate the relative importance of the three goals. These inputs were then processed using the AHP methodology to derive normalized weights, which were subsequently used in the WGP model. The use of AHP not only ensures methodological rigor in weight derivation but also allows for the integration of qualitative judgments in a quantitative optimization framework.Therefore, the weighting scheme is justified as it is derived from a structured decision-making process that is widely validated in the literature and appropriate for the problem context. The process begins with defining the objective, identifying criteria and subcriteria, and constructing a hierarchy. Next, pairwise comparisons between criteria are made using a relative importance scale, which typically ranges from 1 (equal importance) to 9 (extreme importance). These comparisons are used to create a pairwise comparison matrix from which the weights of the criteria are calculated - often by normalizing the matrix and calculating the average of each row. Finally, a consistency ratio is calculated to ensure that the judgments are consistent. If this ratio is acceptable (usually less than 0.1), the resulting weights can be used in the decision-making process. Here, we weighted the three objectives according to expert opinion and formed optimal portfolios based on these results.

Author action: We updated the manuscript by adding these description.

The above description added to manuscript and highlighted in yellow.

________________________________________

Comment # 4: While the integration of sentiment analysis and LSTM for portfolio optimization is innovative, the authors should more explicitly compare their approach with traditional portfolio optimization methods to highlight the added value of their model.

Author response: Thank you for your valuable comment. We agree that comparing our proposed model with traditional portfolio optimization methods is essential to demonstrate its added value. Accordingly, we have added a new subsection titled “Comparison with Other Portfolio Optimization Model” to Section 6.

In this paper, we tried to present a new model in which we used new neural network models and artificial intelligence tools. In this model, we tried to add a new criterion as the outlook of the possible future price movement to improve the model results. Our proposed model included two types of text and price data, which we calculated the price data using the LSTM model, and for text data, we collected the quarterly reports of DJIA companies from the Yahoo Finance website and extracted the companies' outlook for the next quarter using the Cider artificial intelligence tool. To convert this text data into a score, we used the FinBERT model and performed sentiment analysis on the outlooks, and considering that the output of the FinBERT model was a vector (positive, neutral, negative), we used the AHP method to convert this vector into a score and finally extracted the outlook score, this process can be seen in Figure 5. In fact, we tried to present a State-of-the-Art model. The topic of sentiment analysis in most previous models has been that first, sentiment analysis was performed on Twitter data, websites, and social networks, and second, the results were used as one of the features for price prediction. While in our study, we have used data from companies' quarterly reports with an innovative approach. Among the previous studies, the following can be mentioned:

Colasanto et al. [135] In one study, sentiment scores, particularly polarity values, were incorporated into stock price forecasting by introducing them as an additional “view” in the Black-Letterman framework. These scores were extracted from textual analysis of Financial Times articles and were associated with various events, both favorable and unfavorable, that affected specific stocks. The key distinction of the present study is in the application of the FinBERT model, which is used not only as a predictive feature but also to extract insights into future performance from corporate disclosures. Unlike previous approaches that used sentiment as a direct input for price forecasting, our method uses FinBERT to assess the outlook of companies, thereby informing investment decisions with a more interpretative and qualitative layer. Leow et al. [136] introduced two novel models SAW and SMPT which incorporate real-time market sentiment derived from Twitter data, analyzed using BERT. These models adjust portfolio allocation weights based on the extracted sentiment signals. To optimize their performance, genetic algorithms are employed with objectives such as maximizing cumulative returns and minimizing volatility. In contrast, the present study relies on sentiment extracted from corporate quarterly reports to assess future outlooks, offering a more fundamental and document-based approach to sentiment analysis. Day and Li [137] examined the influence of various financial information sources on investment decisions and explored how deep learning techniques can enhance the accuracy of financial news classification. Their empirical findings demonstrated that the type of financial source significantly affects investor behavior and decision-making outcomes, and that classification performance benefits from the application of deep learning models. However, unlike the current study, their research did not address portfolio optimization; instead, it focused solely on analyzing the effects of information quality and classification on investment behavior. Fatouros et al. [138] conducted a pioneering study exploring the capabilities of large language models specificall

Attachment Submitted filename: Authors Response Files.docx

# Peer review: Decision Letter 1

**DOI:** 10.1371/journal.pone.0335036.r003

**Authors:** Junhuan Zhang, Junhuan Zhang

19 Aug 2025

PONE-D-25-02047R1A Multi-Objective Portfolio Optimization Model Incorporating Sentiment Analysis of Quarterly Reports and LSTM-based Price PredictionPLOS ONE

Dear Dr. Sadjadi,

Thank you for submitting your manuscript to PLOS ONE. After careful consideration, we feel that it has merit but does not fully meet PLOS ONE’s publication criteria as it currently stands. Therefore, we invite you to submit a revised version of the manuscript that addresses the points raised during the review process.

Please submit your revised manuscript by Oct 03 2025 11:59PM. If you will need more time than this to complete your revisions, please reply to this message or contact the journal office at plosone@plos.org. When you're ready to submit your revision, log on to https://www.editorialmanager.com/pone/ and select the 'Submissions Needing Revision' folder to locate your manuscript file.

Please include the following items when submitting your revised manuscript:

- A rebuttal letter that responds to each point raised by the academic editor and reviewer(s). You should upload this letter as a separate file labeled 'Response to Reviewers'.
- A marked-up copy of your manuscript that highlights changes made to the original version. You should upload this as a separate file labeled 'Revised Manuscript with Track Changes'.
- An unmarked version of your revised paper without tracked changes. You should upload this as a separate file labeled 'Manuscript'.

If you would like to make changes to your financial disclosure, please include your updated statement in your cover letter. Guidelines for resubmitting your figure files are available below the reviewer comments at the end of this letter.

If applicable, we recommend that you deposit your laboratory protocols in protocols.io to enhance the reproducibility of your results. Protocols.io assigns your protocol its own identifier (DOI) so that it can be cited independently in the future. For instructions see: https://journals.plos.org/plosone/s/submission-guidelines#loc-laboratory-protocols. Additionally, PLOS ONE offers an option for publishing peer-reviewed Lab Protocol articles, which describe protocols hosted on protocols.io. Read more information on sharing protocols at https://plos.org/protocols?utm_medium=editorial-email&utm_source=authorletters&utm_campaign=protocols.

We look forward to receiving your revised manuscript.

Kind regards,

Junhuan Zhang, PhD

Academic Editor

PLOS ONE

Journal Requirements:

If the reviewer comments include a recommendation to cite specific previously published works, please review and evaluate these publications to determine whether they are relevant and should be cited. There is no requirement to cite these works unless the editor has indicated otherwise.

Please review your reference list to ensure that it is complete and correct. If you have cited papers that have been retracted, please include the rationale for doing so in the manuscript text, or remove these references and replace them with relevant current references. Any changes to the reference list should be mentioned in the rebuttal letter that accompanies your revised manuscript. If you need to cite a retracted article, indicate the article’s retracted status in the References list and also include a citation and full reference for the retraction notice.

[Note: HTML markup is below. Please do not edit.]

Reviewers' comments:

Reviewer's Responses to Questions

**Comments to the Author**

1. If the authors have adequately addressed your comments raised in a previous round of review and you feel that this manuscript is now acceptable for publication, you may indicate that here to bypass the “Comments to the Author” section, enter your conflict of interest statement in the “Confidential to Editor” section, and submit your "Accept" recommendation.

Reviewer #1: All comments have been addressed

**********

2. Is the manuscript technically sound, and do the data support the conclusions?

The manuscript must describe a technically sound piece of scientific research with data that supports the conclusions. Experiments must have been conducted rigorously, with appropriate controls, replication, and sample sizes. The conclusions must be drawn appropriately based on the data presented.

Reviewer #1: Yes

**********

3. Has the statistical analysis been performed appropriately and rigorously?

Reviewer #1: Yes

**********

4. Have the authors made all data underlying the findings in their manuscript fully available?

The PLOS Data policy requires authors to make all data underlying the findings described in their manuscript fully available without restriction, with rare exception (please refer to the Data Availability Statement in the manuscript PDF file). The data should be provided as part of the manuscript or its supporting information, or deposited to a public repository. For example, in addition to summary statistics, the data points behind means, medians and variance measures should be available. If there are restrictions on publicly sharing data—e.g. participant privacy or use of data from a third party—those must be specified.

Reviewer #1: No

**********

5. Is the manuscript presented in an intelligible fashion and written in standard English?

PLOS ONE does not copyedit accepted manuscripts, so the language in submitted articles must be clear, correct, and unambiguous. Any typographical or grammatical errors should be corrected at revision, so please note any specific errors here.

Reviewer #1: Yes

**********

6. Review Comments to the Author

Please use the space provided to explain your answers to the questions above. You may also include additional comments for the author, including concerns about dual publication, research ethics, or publication ethics. (Please upload your review as an attachment if it exceeds 20,000 characters)

Reviewer #1: The authors have comprehensively addressed all previous reviewer comments with rigorous revisions. The manuscript now demonstrates significantly improved clarity, methodological transparency, and scholarly rigor. However, some minor formatting items need to be addressed before publication:

Table 13: use the same number of decimal places in every column.

Table 12: be consistent with comma separators—include them for all numbers ≥1,000.

Table 11: use uniform line weights for the three horizontal rules.

Figure 6: distinguish the two curves by adding one solid and one dashed line style.

Please proof-read the entire manuscript once more to ensure consistent wording and formatting throughout.

**********

7. PLOS authors have the option to publish the peer review history of their article (what does this mean?). If published, this will include your full peer review and any attached files.

If you choose “no”, your identity will remain anonymous but your review may still be made public.

**Do you want your identity to be public for this peer review?**  For information about this choice, including consent withdrawal, please see our Privacy Policy.

Reviewer #1: No

**********

[NOTE: If reviewer comments were submitted as an attachment file, they will be attached to this email and accessible via the submission site. Please log into your account, locate the manuscript record, and check for the action link "View Attachments". If this link does not appear, there are no attachment files.]

While revising your submission, please upload your figure files to the Preflight Analysis and Conversion Engine (PACE) digital diagnostic tool, https://pacev2.apexcovantage.com/. PACE helps ensure that figures meet PLOS requirements. To use PACE, you must first register as a user. Registration is free. Then, login and navigate to the UPLOAD tab, where you will find detailed instructions on how to use the tool. If you encounter any issues or have any questions when using PACE, please email PLOS at figures@plos.org. Please note that Supporting Information files do not need this step.

# Author comment: Author response to Decision Letter 2

**DOI:** 10.1371/journal.pone.0335036.r004

23 Aug 2025

Dear Reviewer,

I would like to express my sincere gratitude for your constructive and insightful feedback on the previous version of the manuscript. I am pleased to inform you that all the points you raised have been thoroughly addressed, and the revisions have been made accordingly. The changes are highlighted in yellow in the updated version for your ease of reference.

The following revisions have been implemented as per your suggestions:

Table 13: The number of decimal places has been made consistent across all columns.

Table 12: Comma separators have been applied uniformly for all numbers greater than or equal to 1,000.

Table 11: The line weights of the three horizontal rules have been made uniform.

Figure 6: The two curves have been distinguished by using one solid line and one dashed line style.

Additionally, the entire manuscript has been proofread once again to ensure consistency in wording and formatting throughout.

Please kindly review the revised manuscript. If any further adjustments or clarifications are needed, I am more than happy to make additional changes.

Thank you for your time and consideration.

Best regards,

Prof. Seyed Jafar Sadjadi

The mentioned changes have been highlighted in yellow in the entire manuscript.

# Peer review: Decision Letter 2

**DOI:** 10.1371/journal.pone.0335036.r005

**Authors:** Junhuan Zhang, Junhuan Zhang, Junhuan Zhang

6 Oct 2025

A Multi-Objective Portfolio Optimization Model Incorporating Sentiment Analysis of Quarterly Reports and LSTM-based Price Prediction

PONE-D-25-02047R2

Dear Dr. Sadjadi,

We’re pleased to inform you that your manuscript has been judged scientifically suitable for publication and will be formally accepted for publication once it meets all outstanding technical requirements.

Within one week, you’ll receive an e-mail detailing the required amendments. When these have been addressed, you’ll receive a formal acceptance letter and your manuscript will be scheduled for publication.

An invoice will be generated when your article is formally accepted. Please note, if your institution has a publishing partnership with PLOS and your article meets the relevant criteria, all or part of your publication costs will be covered. Please make sure your user information is up-to-date by logging into Editorial Manager at Editorial Manager® and clicking the ‘Update My Information' link at the top of the page. For questions related to billing, please contact billing support.

If your institution or institutions have a press office, please notify them about your upcoming paper to help maximize its impact. If they’ll be preparing press materials, please inform our press team as soon as possible -- no later than 48 hours after receiving the formal acceptance. Your manuscript will remain under strict press embargo until 2 pm Eastern Time on the date of publication. For more information, please contact onepress@plos.org.

Kind regards,

Junhuan Zhang, PhD

Academic Editor

PLOS ONE

Additional Editor Comments (optional):

Reviewers' comments:

Reviewer's Responses to Questions

**Comments to the Author**

1. If the authors have adequately addressed your comments raised in a previous round of review and you feel that this manuscript is now acceptable for publication, you may indicate that here to bypass the “Comments to the Author” section, enter your conflict of interest statement in the “Confidential to Editor” section, and submit your "Accept" recommendation.

Reviewer #1: All comments have been addressed

**********

2. Is the manuscript technically sound, and do the data support the conclusions?

The manuscript must describe a technically sound piece of scientific research with data that supports the conclusions. Experiments must have been conducted rigorously, with appropriate controls, replication, and sample sizes. The conclusions must be drawn appropriately based on the data presented.

Reviewer #1: Yes

**********

3. Has the statistical analysis been performed appropriately and rigorously?

Reviewer #1: Yes

**********

4. Have the authors made all data underlying the findings in their manuscript fully available?

The PLOS Data policy requires authors to make all data underlying the findings described in their manuscript fully available without restriction, with rare exception (please refer to the Data Availability Statement in the manuscript PDF file). The data should be provided as part of the manuscript or its supporting information, or deposited to a public repository. For example, in addition to summary statistics, the data points behind means, medians and variance measures should be available. If there are restrictions on publicly sharing data—e.g. participant privacy or use of data from a third party—those must be specified.

Reviewer #1: No

**********

5. Is the manuscript presented in an intelligible fashion and written in standard English?

PLOS ONE does not copyedit accepted manuscripts, so the language in submitted articles must be clear, correct, and unambiguous. Any typographical or grammatical errors should be corrected at revision, so please note any specific errors here.

Reviewer #1: Yes

**********

6. Review Comments to the Author

Please use the space provided to explain your answers to the questions above. You may also include additional comments for the author, including concerns about dual publication, research ethics, or publication ethics. (Please upload your review as an attachment if it exceeds 20,000 characters)

Reviewer #1: I would like to thank the authors for their thorough revisions in response to my previous comments. I have reviewed the revised manuscript and the point-by-point response letter. All of my concerns have been adequately addressed, and the corresponding changes in the manuscript have significantly improved the clarity and quality of the work.

**********

7. PLOS authors have the option to publish the peer review history of their article (what does this mean?). If published, this will include your full peer review and any attached files.

If you choose “no”, your identity will remain anonymous but your review may still be made public.

**Do you want your identity to be public for this peer review?**  For information about this choice, including consent withdrawal, please see our Privacy Policy.

Reviewer #1: No

**********

# Editor report: Acceptance letter

**DOI:** 10.1371/journal.pone.0335036.r006

**Authors:** Junhuan Zhang, Junhuan Zhang, Junhuan Zhang

PONE-D-25-02047R2

PLOS ONE

Dear Dr. Sadjadi,

I'm pleased to inform you that your manuscript has been deemed suitable for publication in PLOS ONE. Congratulations! Your manuscript is now being handed over to our production team.

At this stage, our production department will prepare your paper for publication. This includes ensuring the following:

* All references, tables, and figures are properly cited

* All relevant supporting information is included in the manuscript submission,

* There are no issues that prevent the paper from being properly typeset

You will receive further instructions from the production team, including instructions on how to review your proof when it is ready. Please keep in mind that we are working through a large volume of accepted articles, so please give us a few days to review your paper and let you know the next and final steps.

Lastly, if your institution or institutions have a press office, please let them know about your upcoming paper now to help maximize its impact. If they'll be preparing press materials, please inform our press team within the next 48 hours. Your manuscript will remain under strict press embargo until 2 pm Eastern Time on the date of publication. For more information, please contact onepress@plos.org.

You will receive an invoice from PLOS for your publication fee after your manuscript has reached the completed accept phase. If you receive an email requesting payment before acceptance or for any other service, this may be a phishing scheme. Learn how to identify phishing emails and protect your accounts at https://explore.plos.org/phishing.

If we can help with anything else, please email us at customercare@plos.org.

Thank you for submitting your work to PLOS ONE and supporting open access.

Kind regards,

PLOS ONE Editorial Office Staff

on behalf of

Dr. Junhuan Zhang

Academic Editor

PLOS ONE
